# Association between algal productivity and phycosphere composition in an outdoor *Chlorella sorokiniana* reactor based on multiple longitudinal analyses

**DOI:** 10.1111/1751-7915.13591

**Published:** 2020-05-25

**Authors:** Seth A. Steichen, Song Gao, Peter Waller, Judith K. Brown

**Affiliations:** ^1^ School of Plant Sciences The University of Arizona 1140 E South Campus Dr Tucson AZ 85721 USA; ^2^ Pacific Northwest National Laboratory 1529 West Sequim Bay Road Sequim WA 98382 USA; ^3^ Biosystems Engineering The University of Arizona 1177 E 4th St Tucson AZ 85721 USA

## Abstract

Microalgae as a biofuel source are of great interest. Bacterial phycosphere inhabitants of algal cultures are hypothesized to contribute to productivity. In this study, the bacterial composition of the *Chlorella sorokiniana* phycosphere was determined over several production cycles in different growing seasons by 16S rRNA gene sequencing and identification. The diversity of the phycosphere increased with time during each individual reactor run, based on Faith’s phylogenetic diversity metric *versus* days post‐inoculation (*R* = 0.66, *P* < 0.001). During summer months, *Vampirovibrio chlorellavorus*, an obligate predatory bacterium, was prevalent. Bacterial sequences assigned to the Rhizobiales, Betaproteobacteriales and Chitinophagales were positively associated with algal biomass productivity. Applications of the general biocide, benzalkonium chloride, to a subset of experiments intended to abate *V. chlorellavorus* appeared to temporarily suppress phycosphere bacterial growth, however, there was no relationship between those bacterial taxa suppressed by benzalkonium chloride and their association with algal productivity, based on multinomial model correlations. Algal health was approximated using a model‐based metric, or the ‘Health Index’ that indicated a robust, positive relationship between *C. sorokiniana* fitness and presence of members belonging to the Burholderiaceae and Allorhizobium–Neorhizobium–Pararhizobium–Rhizobium clade. Bacterial community composition was linked to the efficiency of microalgal biomass production and algal health.

## Graphical abstract

Microalgae can be efficiently cultivated to produce biomass suitable for conversion to liquid biofuels. Long term experiments conducted over multiple growing seasons in the arid Southwest region of the United States revealed significant associations between algal productivity and the bacterial members of the culture phycospheres. These species offer targets for improving algal biomass yields in mass cultivation.

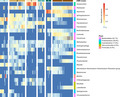

## Introduction

Liquid biofuel has shown great potential to replace a large portion of the global demand for portable energy sources by capturing atmospheric carbon through the process of photosynthesis (Hu *et al*., 2008). Many green microalgae strains can be readily cultivated for biomass feedstock production (Richmond, 2004). As single‐celled organisms, they are more efficient than their more complex terrestrial plant relatives (Metting, 1996). While bioethanol crops have been shown to compete with essential food crops by utilizing precious arable land resources (Wigmosta *et al*., 2011), algae can be grown on marginal lands, achieve high rates of growth, and produce high energy density with some species capable of accumulating as much as 80% of their total biomass as lipids (Hu *et al*., 2008; Singh *et al*., 2011). Large amounts of biomass are required to make microalgal biofuel programmes economically feasible, making open outdoor reactors among the most cost‐effective growth systems, despite certain disadvantages, compared to closed and/or indoor reactors (Jorquera *et al*., 2010). Open systems also provide excellent platforms for research and discovery because they are exposed to a wide spectrum of abiotic and biotic environmental parameters, of which at least a subset is thought to contribute favourably to algal growth.

Several locations in the southwest region of the United States were strategically selected as United States Department of Energy (DOE) testbed sites to evaluate the economic feasibility of biomass production based on factors such as abundant sunlight, heat and predicted maximum growth rates (Wigmosta *et al*., 2011). In previous studies, a field isolate of the green microalgae, *Chlorella sorokiniana* (Shihira and Krauss, 1965) designated DOE strain 1412 (Neofotis *et al*., 2016), exhibited a maximum specific growth rate of 5.9 day^−1^ at 36°C, with significant lipid accumulation (Huesemann *et al*., 2016). As a result, DOE 1412 was prioritized as a primary high‐temperature test strain for outdoor production, including at the DOE Regional Algal Feedstock Testbed (RAFT) site, The University of Arizona, Tucson, AZ, where temperatures commonly exceed 33°C during the summer months. During a three‐year period from 2015 to 2018, the RAFT project conducted cultivation trials under different seasonal, environmental and operational conditions, including a general biocide treatment to ameliorate the effects of a persistent predatory bacterial pathogen of DOE 1412. (Park *et al*., 2018; Steichen and Brown, 2019). The overall aim of RAFT was to optimize the productivity of selected microalgal strains in arid regions through interdisciplinary approaches including engineering, modelling and biological sciences (Ogden *et al*., 2019).

Microalgae are known to associate with microorganisms in natural and artificial environments. Prokaryotic bacteria typically represent the most abundant and impactful of these associations (Cole, 1982; Seymour *et al*., 2017). While axenic cultures of algae are desirable for certain research and industrial applications (Vu *et al*., 2018), large‐scale biomass production systems harbour bacteria naturally and may encounter bacterial invasions. Previous studies of mass‐cultured microalgae recognized that algal‐bacterial associations were generally ubiquitous, but considered their contributions to algal growth to be benign or negative (Myers *et al*., 1951; Krauss and Thomas, 1954). Recent studies have established that complex community dynamics occur between algae and bacteria, and span a range of different ecological relationships (Seymour *et al*., 2017). Based on these observations, the ‘phycosphere concept’ has been proposed, as a corollary to the previously recognized ‘plant rhizosphere’, given robust evidence that bacteria respond chemotactically to algae exudates, thereby defining a zone of algal–bacterial interactions (Bell and Mitchell, 1972). The most commonly detectable phycosphere members are often microbial algal pathogens that cause losses in biomass, which is a quantifiable phenotype. Algae in ecosystems from natural marine environments to biomass production facilities encounter indirect growth inhibition and even direct lysis by amoeba, bacteria, fungi, oomycetes and viruses (Mayali and Azam, 2004; Gachon *et al*., 2010; Wang *et al*., 2010; Carney and Lane, 2014; Carney *et al*., 2014). Production cultures of the DOE 1412 strain of *C. sorokiniana* used in the studies reported here, and other *Chlorella* species, have been reported to collapse following attack by the predatory gram‐negative cyanobacterium, *Vampirovibrio chlorellavorus* (Gromov and Mamkaeva, 1972; Ganuza *et al*., 2016; Park *et al*., 2018; Steichen and Brown, 2019). Alternatively, microbes exist as mutualist symbionts with higher plants, often promoting plant health and contributing to growth and reproduction (Lugtenberg and Kamilova, 2009; Nadeem *et al*., 2014). Analogous observations have been reported for microalgal species, including *C. sorokiniana*, which showed significantly higher growth rates in the presence of the diazotrophic, indole‐3‐acetic acid producing *Azospirillum brasilense* and *Bacillus pumilus* bacteria (Amavizca *et al*., 2017), and certain naturally co‐occurring fungi (Watanabe *et al*., 2005). Importantly, microbiome interactions with host phenotypes are often best modelled using abundances of multiple species rather than individual taxon, highlighting the interconnected nature of most biological systems (Sze and Schloss, 2018).

Understanding the contributions of individual taxa and networks of interactions with algae within the phycosphere requires the ability to detect the predominant as well as the less abundant types of microbes. Traditional methods of culturing microbes have been shown to identify as few as 1% of total microbial diversity (Stewart, 2012). The use of culture‐independent sequencing of microbial DNA has become a routine technique, relying on conserved regions in the microbial genome. The 16S ribosomal RNA gene is one of the most commonly used molecular markers to distinguish organisms (Woese, 1987) with ~ 1% sequence divergence indicating different bacterial species (Edgar, 2018). Additional approaches have been developed to facilitate the detection of the most abundant and more rare bacterial species in samples by high‐throughput sequencing of the 16S rRNA gene amplicons, making it possible to determine tens of thousands of individual sequence fragments in a sample (Caporaso *et al*., 2012). Initially, only clustering of sequences representing the different taxa into groups was feasible, based on sequence similarity and referred to as operational taxonomic units (OTUs). However, advancements in computing power and algorithms have permitted the differentiation of individual amplicon sequence variants (ASVs), enabling the detection of maximum ‘signals’ within a data set (Callahan *et al*., 2017; Edgar, 2018).

The parameters recorded and evaluated in this study were pH, algal density, dissolved oxygen, algal growth rate, temperature, precipitation, solar radiation and wind speed. All the parameters were measured and archived for each cultivation cycle of *C. sorokiniana* 1412 in the RAFT reactors. The resultant data were analysed to identify the most relevant parameters or factors influencing the composition of the *C. sorokiniana* phycosphere, with an emphasis on the spatial and temporal patterns in the microbiome community over two consecutive growth seasons. The objective was to parse the microbiome community into groups and identify those having the greatest influence on DOE 1412 health, growth and biomass productivity. Two different sets of experiments were designed and used for determining the composition of bacteria associated with the phycosphere community. The first experimental studies, R41 and R42, were used to conduct replicated, longitudinal analysis of the reactor culture, with twice daily sampling and was used to establish the composition of the ‘baseline phycosphere community’ under optimal growth conditions. The second set of experiments, R10 through R27 (Table S1), were conducted over two growing seasons in paddlewheel and ARID reactors and were sampled every other day to determine the microbiome community composition based on analysis of 16S rRNA gene sequences, for a cross section of RAFT cultures. The goal of the study was to identify patterns potentially linked to different environmental conditions, including those predicted by modelling to contribute most to algal growth and biomass productivity.

## Results

### Baseline community structure analysis

**Fig. 1:**
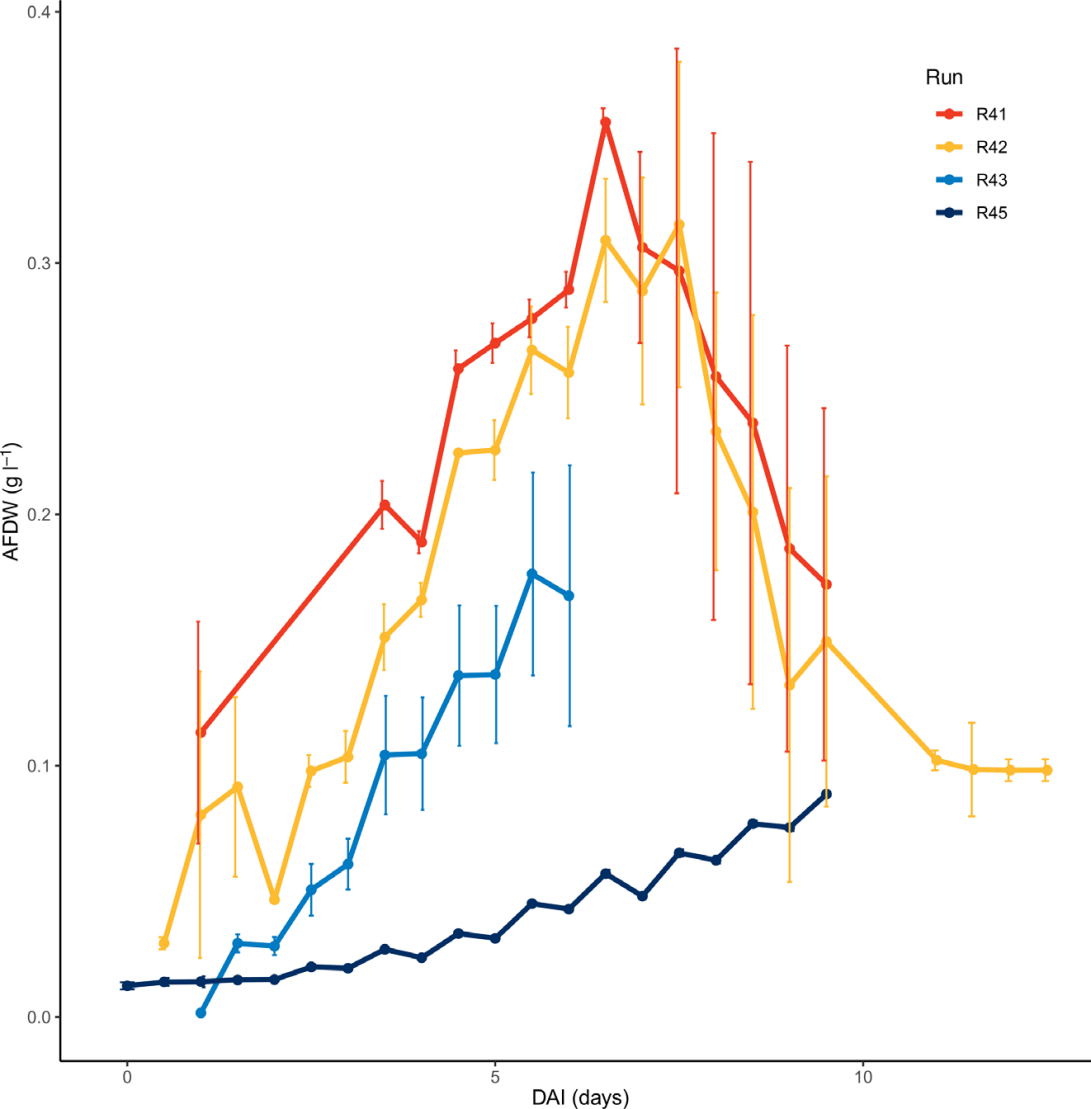
Growth of RAFT baseline experiment cultures. Each line depicts the mean ash‐free dry weight (g l^−1^) concentrations for each duplicate run. Error bars represent standard deviation (*n* = 2). Benzalkonium chloride treatments were applied to R41 and R42 cultures.

The typical development of bacterial phycosphere communities in outdoor DOE1412 cultures was established by analysing the 16S rRNA gene content of samples collected twice daily from paddlewheel reactors operated under standard practices established during the RAFT project. The growth curves of the two benzalkonium‐treated experiments, R41 and R42, were similar to each other and showed high growth rates than the untreated experiments. The variance of the observations of biological replicates within the experiments was highest during the death phase (Fig. 1). Average temperatures of outdoor cultures declined during subsequent experiments while pH was continuously held near 8 by carbon dioxide injections (Table S1). The diversity of phycosphere bacteria increased with time, post‐inoculation, irrespective of benzalkonium chloride treatment (*R* = 0.66, *P* < 0.001) or not (*R* = 0.47, *P* < 0.001; Fig. S1).

**Fig. 2:**
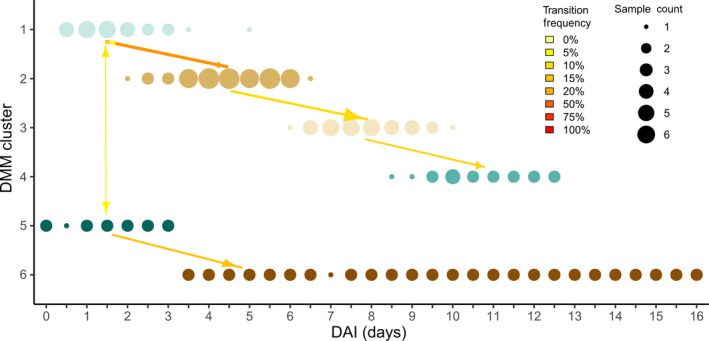
DMM cluster group together based on cultivation time in the outdoor RAFT reactor. Circles are coloured by their DMM cluster assignment and sizes are scaled by the number of samples collected at the same time point for the same DMM cluster. Number of samples collected from each of the same reactors on the subsequent day is indicated by vector lines between the DMM clusters.

The phycosphere samples collected during baseline experiments were grouped into six clusters based on the similarity of their ASV frequencies using a Dirichlet multinomial mixture (DMM) modelling approach with lowest Laplace approximation (Fig. S2; Holmes *et al*., 2012). The clusters show a correspondence to the amount of time culture samples were exposed to outdoor cultivation (Fig. 2). Cluster 1 through 4 represented distinct phases of phycosphere development as indicated by samples collected on consecutive days from the same reactor transitioning across these clusters over the course of cultivation (Fig. 2). All the samples in clusters 5 and 6 were collected during experiment R45, which also demonstrated the most distinctly different growth pattern from the other replicates (Fig. 1). The samples in clusters 1 and 2 corresponded with the early algal growth phase, while clusters 3 and 4 consisted of samples collected after day 6, when the culture algal cell densities began to decline. Thus, the changes in algal growth pattern appeared to be correlated with the composition of bacteria in the phycosphere.

**Fig. 3:**
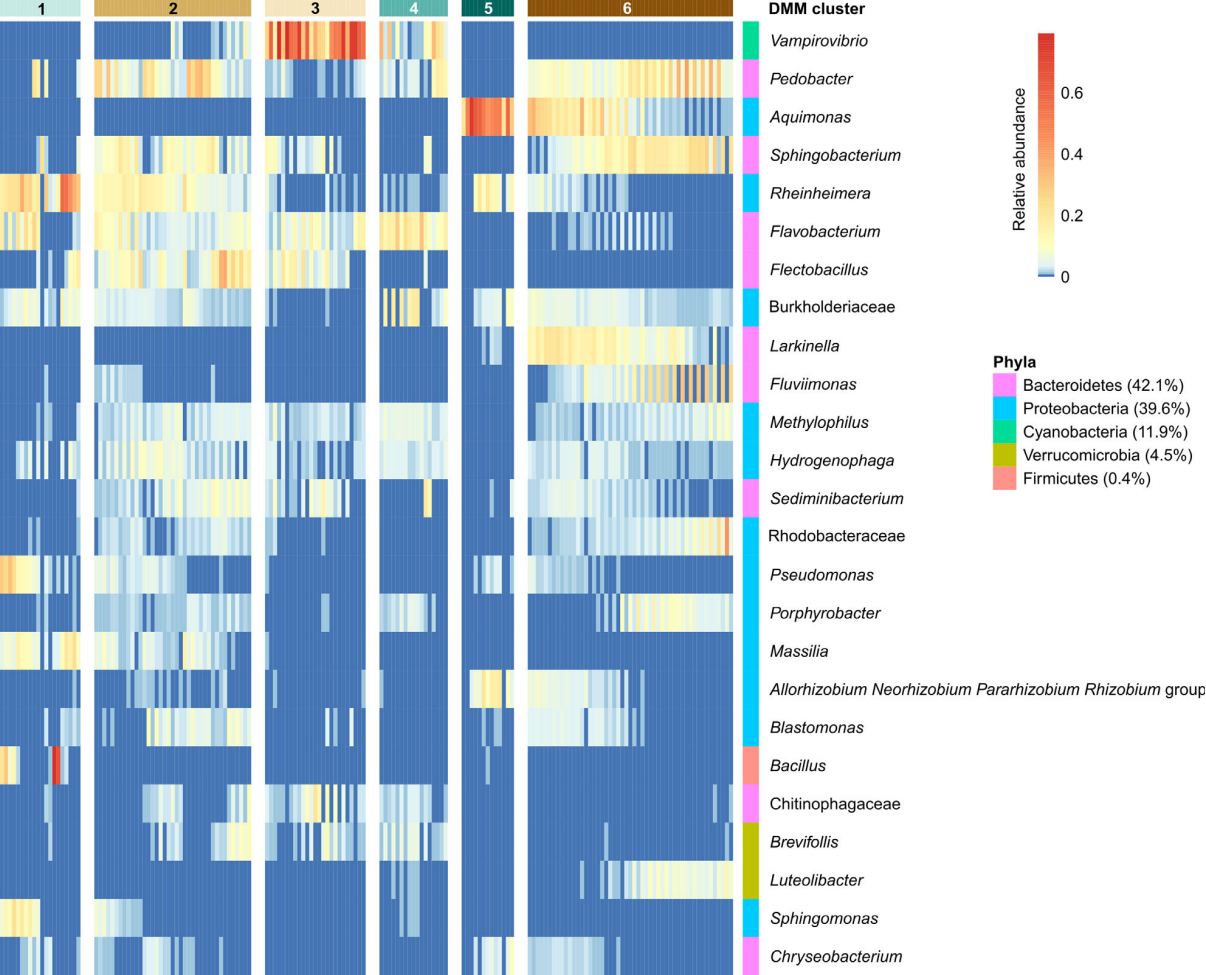
DMM clustering of RAFT baseline experiment 16S ribosomal RNA gene sequence (*n* = 165). The heatmap shows the relative abundance of the 25 most common genera identified for each sample. Columns represent samples, which are grouped by the respective DMM cluster. Rows are labelled based on genus‐level identification, or by family‐level identification when classification was uncertain, with the corresponding phyla annotation indicated by the differently coloured columns.

The predominant phyla represented in phycosphere samples were identified as members of the Bacteroidetes and Proteobacteria, comprising 42% and 40% of the total 16S rRNA gene sequences, respectively. The most abundant ASV was identified as the genus, *Vampirovibrio*. The most commonly occurring sequence variant among those classified within the *Vampirovibrio* matched the full‐length *Vampirovibrio chlorellavorus* 16S rRNA gene sequence, at 100% nucleotide identity, previously determined from the RAFT outdoor reactor samples associated with decline of the *C. sorokiniana* cultures (Park *et al*., 2018; Steichen and Brown, 2019). Samples collected during times corresponding to algal culture decline (Fig. 1) were grouped together into DMM clusters 3 and 4, which were comprised of average relative frequencies of *V. chlorellavorus* at 46% and 11%, respectively (Fig. 3). Conversely, clusters 1 and 2, which corresponded to algal growth phase, were typified by relatively higher proportions of bacteria from the *Rheinheimera*, *Pseudomonas*, *Massilia* and *Bacillus* compared to those samples collected from dying cultures.

### Bacterial correlations with phycosphere variables during two growing seasons

Associations between bacterial phycosphere composition and algal culture growth parameters were identified by examining 16S rRNA gene data from samples collected during the baseline experiments together with a broad collection from cultures grown during seasons of optimal daytime growth temperatures (~ 25–35°C) for *C*. sorokiniana DOE 1412 (Fig. S3). Consistently observed patterns of phycosphere membership across this larger data set represented the most robust interactions that persisted through all the variant environmental conditions that would be encountered during long‐term cultivation efforts in the Southwestern United States. Bacterial ASV correlations with algal concentration, measured by ash‐free dry weight (AFDW), showed more variation than correlations with the other seven parameters in a multinomial regression model (Fig. S4). However, AFDW is an instantaneous measurement that is not indicative of algal population growth rate as can be seen when compared to the concentration of oxygen in the media, which is the direct result of active respiration by algae cells. Biomass productivity is a measurement of the change in algal concentration over time, providing a better reflection of cell division or loss as indicated by its close correlation to dissolved oxygen (Fig. S5). Bacterial ASVs correlated with biomass productivity were assigned predominantly to the orders Rhizobiales, Betaproteobacteriales and Chitinophagales (Fig. S6). The Rhizobiales and Betaproteobacteriales demonstrated a positive relationship with algal biomass productivity relative to the known *C. sorokiniana* pathogen, *Vampirovibrio chlorellavorus*. These relationships were defined primarily by samples collected from declining algal cultures, indicating that a negative log ratio of either of these orders to Vampirovibrionales was consistently associated with algal culture crash (Fig. S7). The frequencies of representatives in the order, Bacillales and order, Pseudomonadales were found to be negatively correlated with days after inoculation of the algal cultures outdoors (Fig. S8), in agreement with the developmental patterns identified by DMM cluster analysis of the baseline experiments (Fig. 3).

Bacterial ASVs assigned to taxa with previously reported relationships to algal growth as well as patterns uncovered during the present study were further investigated by comparing their changing relative abundances across measured biomass productivities in reference to an ASV (6e9594a6005cc8cede0ca7532ff77bfa) assigned to the Sphingobacteriaceae family (Fig. S9). This sequence was selected due to its observed ubiquity in the data set, representing 6.2% of all sequence reads and being detected in 81.0% of samples collected. The ASV was also not significantly correlated with biomass productivity by multinomial model analysis, with a differential of −0.06 near the middle of the distribution for the measure (Table S2). During the RAFT experiments, the orders Rhodobacterales and Vampirovibrionales were both negatively correlated with biomass productivity according to multinomial modelling results (Table S2) and the pattern was further supported by linear regression (Fig. S9b and d). Burkholderiaceae ASV (21c587e98537024a404d6f041c0fb693) and an ASV matching to the previously described algae growth‐promoting *Rhizobium sp*. (Kim *et al*., 2014) were selected as examples of possible algal mutualist in the RAFT experiments, but failed to produce significant linear correlations with biomass productivity (Fig. S9a and c).

The most significant relationships between bacterial ASVs and the weather variables; precipitation, solar radiation and wind speed were generally negative, as indicated by the distributions of outliers in Figure S4. During the relatively rare periods of precipitation at the study site, the most depleted bacterial orders in the phycosphere samples included Betaproteobacteriales, Chitinophagales and Sphingomonadles that together accounted for 11 of the 22 statistical outliers in the multinomial model (Table S2). Those statistical outliers of bacteria associated with wind speed were distributed among 18 different taxonomic orders, with the Rhizobiales being the most commonly depleted during high winds, with five ASVs of the order among the statistical outliers. The extreme solar radiation experienced at the test site was linked negatively with a variety of bacterial taxonomies, but most commonly with the orders Betaproteobacteriales, Chitinophagales and Rhizobiales (Table S2).

### Biocide treatments were consistently associated with changes in bacterial phycosphere composition

**Fig. 4:**
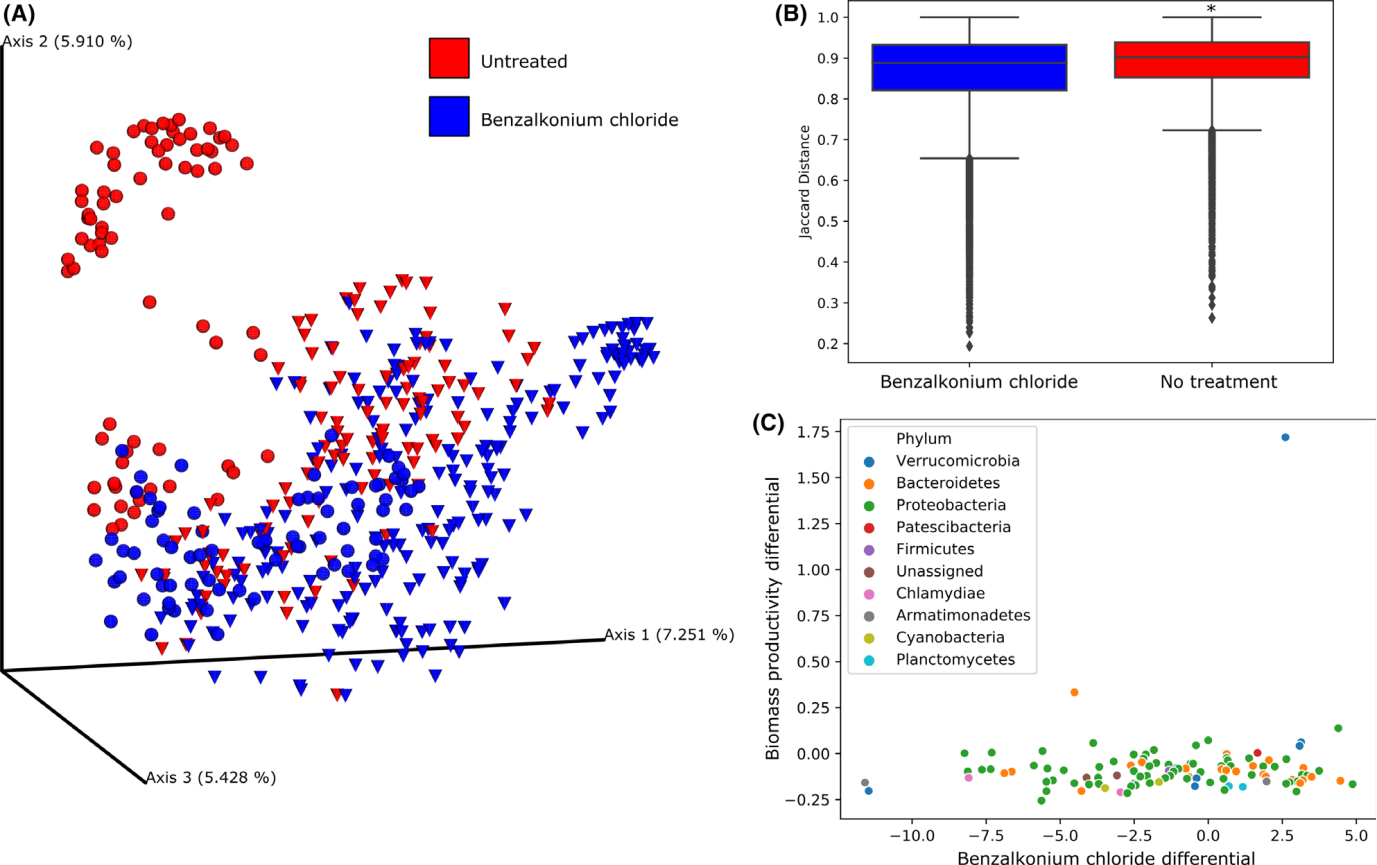
Changes in bacterial community structure associated with benzalkonium chloride treatments. The Jaccard distances between samples collected during RAFT experiments (*n* = 476) are displayed in an (A) principal coordinate analysis (PCoA) plot with those collected following benzalkonium chloride treatment coloured blue and untreated samples coloured red. Samples collected during the baseline experiments are denoted by spheres (*n* = 148) and survey experiments by cones (*n* = 328). Beta diversity comparisons between samples from Benzalkonium chloride‐treated cultures and untreated are displayed in (B) boxplots as the distance to the centroid of treated samples was determined to be significant by PERMANOVA analysis (*P* = 0.001). The ASVs determined to be significantly differentially abundant between treatments by ANCOM analysis (*n* = 107) are (C) plotted according to multinomial model differential values for biomass productivity and BAC treatment variables.

Benzalkonium chloride (BAC) was applied to treat infections of project cultures by *Vampirovibrio chlorellavorus* bacteria. Applications of BAC reduced accumulation of the pathogen and increased algal growth duration (Steichen and Brown, 2019). The bacterial phycospheres of samples collected from treated cultures shared more similarity to each other than to untreated culture phycospheres in a principal coordinate analysis of their Jaccard beta diversities (Fig. 4A). This observation was supported by a significant difference between the sample groups (*P* = 0.001) when tested by the PERMANOVA method (Fig. 4B; Table S3). Among the total tested ASVs (*n* = 914), 107 were determined to be present at significantly different relative abundances between the BAC‐treated and BAC‐untreated sample groups based on an ANCOM statistical analysis. The majority of the differentially abundant ASVs shared a negative association with the BAC treatment (*n* = 65) according to their multinomial model differential values, but these bacteria did not show a strong correspondence to biomass productivity (Fig. 4C).

### Relationships between bacteria and algal culture health

**Fig. 5:**
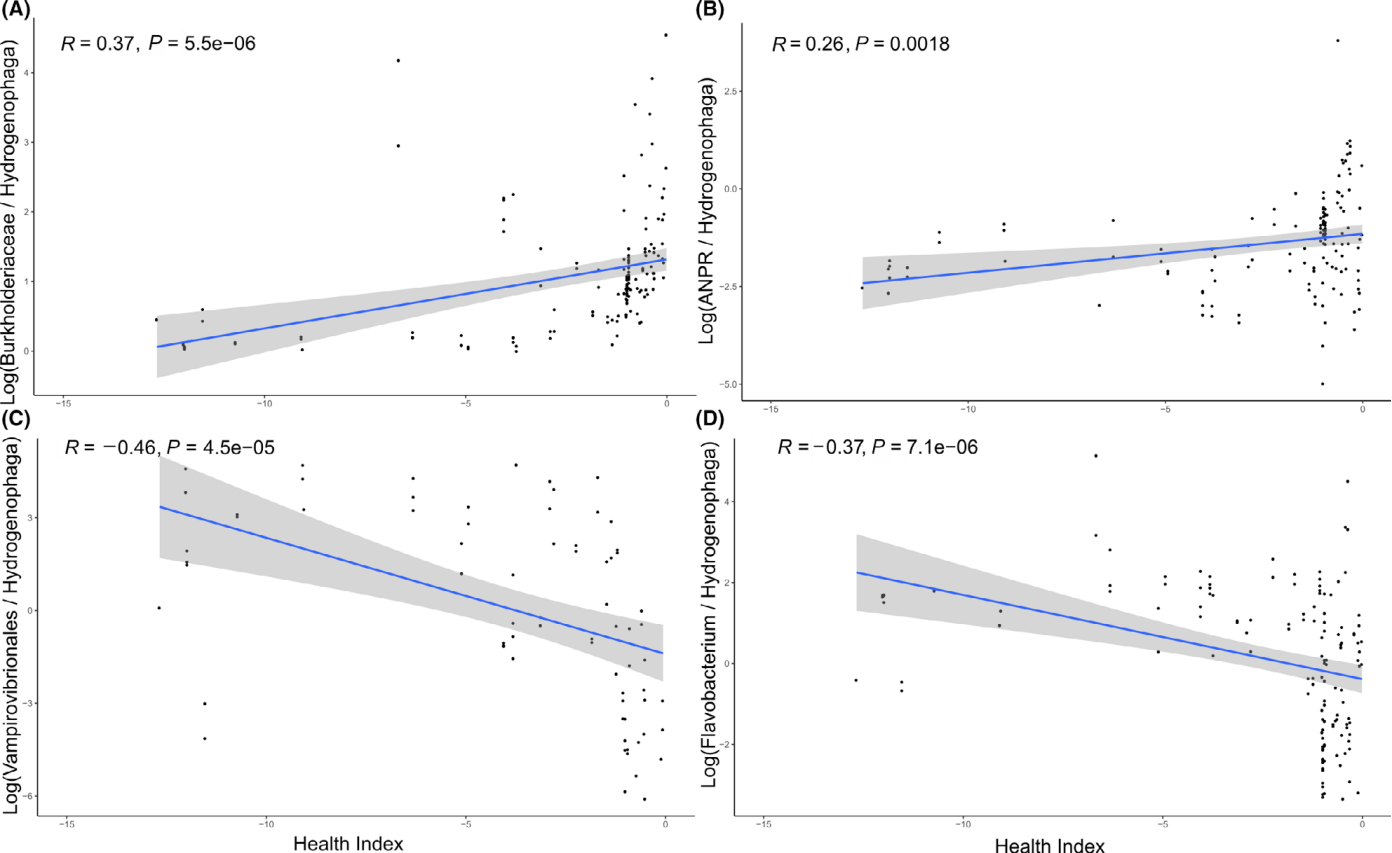
Log ratio relationships of selected bacterial taxa to algal Health Index (HI). The log ratios of the relative abundance of (A) Burkholderiaceae, (B) Allorhizobium–Neorhizobium–Pararhizobium–Rhizobium (ANPR), (C) Vampirovibrionales and (D) Flavobacterium to Hydrogenophaga for each sample are plotted by their corresponding algal HI measure. Lines and grey areas represent linear regression and 95% confidence intervals, respectively, and R fitness and *P* significance values, based on Pearson’s correlation test.

During the replicated baseline experiments, algal culture health was quantified by a Health Index (HI) metric, which compared observed growth rates to simulated growth rates calculated using a model that includes minimal bacterial effects. More than 93% of the observed growth rates were lower than their corresponding simulated growth rate (Fig. S10a), meaning that greater HI scores approaching zero were indicative of healthy cultures approaching the predicted growth rate. The HI was highest and most stable two to four days following culture inoculation across the replicates (Fig. S10b) and was most negative during sampling times with low or negative observed growth rates (Fig. S10c). The negative HI values during the later time points corresponded again with the declining algal cultures during the second half of the baseline experiments. There were fewer overall ASVs significantly correlated with HI (Fig. S11) than to biomass productivity, but their taxonomic assignments were similar at the order level (Fig. S6). Correlations between selected bacterial ASVs and HI were more significant than either biomass productivity or AFDW (Fig. S12). Bacterial ASVs from the Burkholderiaceae family and Allorhizobium–Neorhizobium–Pararhizobium–Rhizobium clade were positively correlated with HI with reference to the ubiquitously distributed Hydrogenophaga (Fig. 5A and B), lending further evidence to the possibility of mutualistic promotion of algal growth by these bacteria. The negative correlations of order, Vampirovibrionales and Flavobacterium ASVs to HI demonstrated that the metric identified previously known and putative algal antagonists (Fig. 5C and D).

## Discussion

Culture‐independent methods have led to a deeper appreciation of the microbes associated with the human gut (Stewart *et al*., 2018), plant rhizosphere (Berendsen *et al*., 2012) and various open or natural environments (Thompson *et al*., 2017). The interpretation of 16S rRNA gene sequence data relies on determination of true sequence variants through the use of de‐noising algorithms followed by assignment to known taxonomic groupings by comparison with sequences in curated databases (Bokulich *et al*., 2018). Assemblages of eubacterial and archaeal organisms (Parada *et al*., 2016) in the phycosphere of experimental, outdoor open algal production reactors (DOE‐RAFT project DE‐EE0006269, The University of Arizona) were identified by comparative analysis of the 16S rRNA gene sequence. Environmental and experimental variables were collected over more than two years of cultivation and correlated with shifts in the bacterial community structure. The associations between bacteria and microalgal biomass productivity and of culture ‘health’ under a scenario of repeated rounds of infection of the algal host by the predatory bacterial pathogen, *V. chlorellavorous*, were investigated to help identify conditions to guide optimization of biomass production in the RAFT reactors. Results demonstrated that the community was dynamic, complex and responsive to several key environmental variables. While the approaches used in this study were able to capture evidence of a fluctuating eubacterial composition associated with the *C. sorokiniana* algal cultures, the specific mechanisms guiding the observed biological fluxes are not yet known. However, it was possible to apply available information about certain closest bacterial relatives obtained from previously studied phycosphere and rhizosphere systems to develop hypotheses for further testing.

The bacterial phycosphere composition changed throughout the different cultivation cycles and over time, with community diversity increasing (Fig. S1), while certain taxa became more or less prevalent than others (Fig. 2). The fixation of gaseous nitrogen into bioavailable ammonium in exchange for organic carbon is one of the most well‐known reactions involving land plants and rhizosphere‐associated microorganisms (Berendsen *et al*., 2012). An analogous phycosphere association has been reported to occur in marine environments where widely distributed prokaryotes that are associated with their unicellular phytoplankton express the *nifH* nitrogenase gene, among other dynamic mutualistic contributions (Thompson *et al*., 2012). The order Rhizobiales, which contains numerous nitrogen‐fixing rhizosphere symbionts, was overrepresented among ASVs significantly associated with algal biomass productivity (Fig. S6). Within the Rhizobiales, bacteria belonging to the genus, *Devosia*, represented two of the ten ASVs whose presence was most closely associated with *C. sorokiniana* growth (Table S4). The genus, *Devosia,* contains members that have been previously isolated from root nodules of aquatic leguminous plants (Rivas *et al*., 2002), suggesting the possibility of a mutualistic partnership with *C. sorokiniana* involving the partitioning of nitrogen for uptake by algae and other phycosphere members. The family, Burkholderiaceae, sequence variants were very highly correlated with biomass productivity and contain known nitrogen‐fixing bacteria (Sawada *et al*., 2003). The abundance of members of the order, Chitinophagales increased after the initial algal growth phase. This pattern was observed in the baseline experiments for samples collected 6 days post‐inoculation of reactors (Figs 2 and 3), and Chitinophagales ASVs were positively correlated with number of days in culture during the seasonal monitoring (Fig. S8). The latter group are related to bacteria capable of consuming chitin, and may be opportunists on other phycosphere bacterial species, or the microalgal cell walls that are composed of chitin and chitosan (Blanc *et al*., 2010).

Plant growth‐promoting rhizobacteria (PGPR) have been shown to be phylogenetically diverse, and illicit increased fitness of their terrestrial plant partners by various mechanisms (Lugtenberg and Kamilova, 2009; Berendsen *et al*., 2012). Several studies have focused on the promotion of algae growth by taxonomically and functionally diverse bacteria. In one example, *Rhizobium spp*. were the major phycosphere constituents for four different green microalgal species, including *Chlamydomonas reinhardtii* P.A. Dangeard 1888, *Chlorella vulgaris* Beyerinck 1890, *Scenedesmus sp*. and *Botryococcus braunii* Kützing (Kim *et al*., 2014). The most commonly occurring full‐length 16S rRNA gene sequence in the study was identified as a *Rhizobium spp*. (EU781656; Kim *et al*., 2014). In the RAFT reactors, an isolate sharing 100% nucleotide identity with this *Rhizobium* spp., ASV (b449f5066387be07ee577a95585d45ca), was present in 39.7% of samples analysed during routine monitoring studies. Also, a second *Rhizobium* spp. (JX255399), following growth in pure culture, was shown to promote the growth of the four algal species following inoculation of the algal cultures (Kim *et al*., 2014). This bacterial 16S rRNA sequence was 100% identical to the ASV, 8ce12e88f6b59bb09494567f0d678092, from the RAFT data set for which it was present in 5.6% of the samples. However, neither the ubiquitous nor the growth‐promoting *Rhizobium* spp. were significantly correlated with biomass productivity, based on coefficients of −0.012 and −0.118, respectively (Table S2), and, the linear regression analysis identified one insignificant positive relationship (Fig. S9c). These observations indicate that while phylogenetically identical bacterial species may apparently occur in different phycospheres, their specific contribution to the community may be specific to habitat and/or algal species composition.

In a recent study, several *Azospirillum* spp. were shown to increase the growth rate of *Chlorella sorokiniana* (Shihira and Krauss, 1965) facilitated by its contribution the hormone, indole‐3‐acetic acid (IAA; De‐Bashan *et al*., 2008). Additional studies showed that algae growth increased following inoculation of cultures with *Bacillus pumilus* (Hernandez *et al*., 2009; Bashan *et al*., 2016; Amavizca *et al*., 2017). The RAFT samples contained seven ASVs that are assigned to the order Azospirillales, and however, they were found to be negatively correlated with biomass productivity. While the sole *Bacillus sp*. ASV identified in the RAFT samples during monitoring had a −0.093 correlation with biomass productivity, two of the six members of the order Bacillales were positively linked to growth. Further, a *Chlorella* spp. culture having a low growth rate has been shown to exhibit increased growth when grown in laboratory cultures to which a bacterial consortium from a fast‐growing algal culture was used to augment productivity. An analysis of the relative abundances of 16S rRNA gene sequences revealed that several *Ruegeria* sp. were enriched in the fast‐growing cultures. While no members of the genus, *Ruegeria* ASVs were identified among the RAFT samples, the order Rhodobacterales to which *Ruegeria* is assigned, were highly correlated with biomass productivity and rapid growth in laboratory cultures (Richter *et al*., 2018).

Bacteria that were negatively associated with algal growth during the RAFT project may have been antagonistic or possibly pathogenic to *C. sorokiniana*, and so are prospective candidates of taxa that could serve as indicators for declining algal culture health. Six ASVs were identified that were significantly negatively associated with *C. sorokiniana* biomass productivity, based on the multinomial model. These were identified as members of the Rhodobacteraceae, order Nomurabacteria and the genera *Pseudohongiella, Luteolibacter, Cytophaga* and *Pseudomonas*. (Table S4). Among them, only the genus, *Cytophaga,* has been robustly linked to algicidal activity and killing of diatom species (Imai *et al*., 1993; Mayali and Azam, 2004), predation on cyanobacteria (Rashidan and Bird, 2001), and accumulation during stationary and death phases of a mixed photobioreactor culture containing members of the family, Scenedesmaceae (Carney *et al*., 2014).

The predatory cyanobacterium, *V. chlorellavorus*, is recognized as a virulent pathogen of several species of *Chlorella* (Coder and Goff, 1986; Soo *et al*., 2015; Ganuza *et al*., 2016). It has been previously shown to be responsible for the death of *C. sorokiniana* cultures in Arizona at the RAFT site (Steichen and Brown, 2019). Experiments carried out in this study showed that the infection cycle could be managed to some extent through applications of the quaternary ammonium complex, benzalkonium chloride (BAC), a general biocide. However, it is not known whether the apparently marginal negative effects on algae growth were directly related to the biocide itself, or indirectly, because of the potential negative effects, it would be expected to have on some or all of the phycosphere bacterial community. In general, there was no significant difference between the biomass productivity of the algal grown in the BAC‐treated reactors or in the untreated (negative) control reactors (Fig. S13a). However, this result bears some uncertainty because during the time in the cultivation season during when reactors were treated with BAC, the outdoor temperatures were higher than several weeks earlier when reactors contained the BAC‐untreated cultures (negative control; Fig. S13b). An additional caveat is that typically higher temperatures result in faster growth and increased susceptibility of C*. sorokiniana* to attack by *V. chlorellavorus*. The hypothesis that algal growth rate was indirectly affected by changes in bacterial community is not supported by the ANCOM results (Fig. 4C) that indicated an overall suppression of the most significantly affected bacteria, albeit the latter ASVs did not show a strong relationship to biomass productivity. The distribution of the taxonomic assignments of ASVs significantly affected by the BAC treatment was similar to the overall data set, except for the bacteria assigned to the phyla, Firmicutes, for which only a single ASV was significantly changed by BAC treatment, despite being the third most commonly assigned taxa in the collective data set (Table S5). The Firmicute bacteria, *Listeria monocytogenes,* encodes an efflux pump that confers resistance to BAC (Kovacevic *et al*., 2016), offering a mechanism by which members the phylum could have feasibly avoided the potentially toxic effects of the BAC treatment.

Taking quantitative measurements of algal health in outdoor reactor cultures is challenging. On a regular basis, algal cultures are exposed to continuously changing environmental conditions, particularly light intensity and water temperature. Consequently, the growth of the algae and microorganisms that comprise the phycosphere and their interactions are dynamic. Also, algal growth is affected by its own properties and capacity to adapt to and flourish in the collective environment. For example, a ‘healthy’ dense algal culture would be expected to grow more slowly than a healthy, low‐density culture in part owing to increased self‐shading. The algal Health Index (HI) computed in this study showed promise for the ability to identify significant bacterial contributors to growth of the target alga. The HI was found to be more sensitive to identifying bacterial correlations in that there were more significant linear correlations between the pathogen *V. chlorellavorus* and the putative mutualist bacteria, compared to the biomass productivity and AFDW (Fig. S12). While the latter two metrics are widely used for assessing industrial biomass production goals, applying the HI calculation developed in this study provided a more accurate depiction of biological fitness of the algal populations analysed here.

The goal of the RAFT project was to develop a global cultivation system for the efficient production of algal biomass. The microbial members of the phycosphere that were identified in this study and associated with algal health are possible targets for further assessment. The means by which they influence algal health, whether directly or indirectly, require further study, including specifically how they function and whether they can be utilized in culture augmentation. Further, some of these taxa may prove to be reliable indicators of productivity for *C. sorokiniana* and for other algal species as well. In the near term, culture‐independent analysis of the entire bacterial community is not well‐suited for monitoring, and so the design of more specific assays is needed to enable their easy adaptation for field use. For example, a qPCR assay could be developed to monitor Rhizobiales using the 16S rRNA gene sequence identified in this study as having a tight association with algal growth. These approaches can feasibly provide the ability to obtain rapid, quantitative measures of algal health for the incorporation into models, along with other parameters, that will sound a warning or an alert indicating a need for intervention. With the increasingly economical costs of PCR amplification and DNA sequencing for routine monitoring and microbiome analysis, the phycosphere community composition can be routinely monitored in reactors and the ‘sentinel ASVs’ easily identified by specific qPCR amplification. Further, modelling cultures with microbiome analyses coupled to algal Health Index can facilitate more complete assessment of algal culture health than is presently achieved by measuring algal density alone.

## Conclusion

Bacterial community composition was linked to the efficiency of microalgal biomass production and algal health in outdoor reactors that are representative of a practical biofuel operation. The Rhizobia and Chitinophagales bacteria identified here represent positive and negative indicators of *C. sorokiniana* culture performance, respectively. These are candidates for further experimentation to determine whether they can be used to adjust culture performance or monitored for predictions of biomass accumulation or loss.

## Experimental procedures

### Algal culture

The *C. sorokiniana* strain DOE1412, previously referred to as NAABB 2412 (Lammers *et al*., 2017), was isolated from surface water at a collection site in Texas, USA [provided by Dr. J. Polle, Brooklyn College] (Huesemann *et al*., 2013). The culture was thereafter maintained in the laboratory on BG‐11 media containing 17.6 mM of NaNO_3_, 0.22 mM of K_2_HPO_4_, 0.03 mM of MgSO_4_·7H_2_O, 0.2 mM of CaCl_2_·2H_2_O, 0.03 mM of citric acid·H_2_O, 0.02 mM of ammonium ferric citrate, 0.002 mM of Na_2_EDTA·2H_2_O, 0.18 mM of Na_2_CO_3_, with the addition of trace metals (Rippka and Herdman, 1992). Cultures were maintained by periodic serial transfer on solid BG‐11 media containing 30 g l^−1^ of agar. For cultivation, laboratory and outdoor reactor cultures were grown in a modified media designed to obtain high yields while minimizing nutrient inputs, referred to as Pecos media (PE07; Lammers *et al*., 2017). The PE07 media contained 1.7 mM of urea ((NH_2_)2CO), 0.05 mM of MgSO_4_·7H_2_O, 0.3 mM of NH_4_H_2_PO_4_, 1.4 mM of Potash (KCl), 0.03 mM of FeCl and BG‐11 trace metal solution. Field experiments were conducted at the University of Arizona outdoor test site (+32°16ʹ49.29ʺ, −110°56ʹ9.82ʺ) in three freshwater reactors, two 762 L traditional paddlewheel (PW) reactors (Crowe *et al*., 2012) and a larger sunken basin style of reactor termed Algae Raceway Integrated Design (ARID; Waller *et al*., 2012).

### Outdoor cultivation and benzalkonium chloride biocide treatment

Reactors were inoculated with laboratory grown DOE 1412 cells at an optical density of 0.2 (OD750), equivalent of between 3∙10^6^ and 5∙10^6^ cells ml^−1^, in PE07 media. Collected biomass was maximized by harvesting 75% of culture volume during exponential algal growth. The algal density set for harvest was OD750 ≥ 1.5 based on growth dynamics observed for DOE1412 in the local conditions (Ogden *et al*., 2019). The water and 1× equivalent media nutrients were immediately replenished. Samples were collected for bacterial phycosphere analysis every other day from selected experiments over a two‐year period. Cultures were scored as dying upon observed decrease in algal cell density over two consecutive days. When *V. chlorellavorous* was considered a risk, the established management practice was the addition of a biocide to abate the attack. To simulate this, one reactor was treated with 2 ppm benzalkonium chloride (BAC) every fourth day, and the other was untreated, and used as the negative control. Biomass productivity was determined based on the ash‐free dry weight (AFDW) (g). The AFDW was used to calculate the areal productivity, which was determined by subtracting biomass concentration (g l^−1^) from that of the previous measured time point, multiplied by the reactor volume, and divided by the surface area and the number of days from the last AFDW measurement, expressed as g m^−2^ day^−1^ (Pedroni *et al*., 2004). A subset of replicated experiments were sampled twice daily for bacterial 16S rRNA gene analysis to establish a baseline of typical phycosphere development at the RAFT experiment site. DOE 1412 biomass was cultivated in duplicate raised paddlewheel reactors during two runs treated with BAC (RAFT 41 and 42), and two more runs without the treatment (RAFT 43 and 45). The baseline experiments were run as batches with addition of nutrients at the beginning of the run and no further harvest and water replenishment cycles.

### Measure of culture health

The health of algal populations has been shown to be partly dependent on its associated phycosphere, both positively and negatively given different compositions. In this study, the difference between the maximum biomass growth rate and the suppressed, observed, biomass growth rate was tested as a measure of culture health. The growth of algae and other phycosphere cells were subject to the constantly varying environmental conditions such as sunlight intensity and water temperature. Moreover, the growth slowed down as the increased biomass density induces more self‐shading. Therefore, the maximum biomass growth rate of a healthy culture was constantly evolving over time. In this study, a biomass growth model (Huesemann *et al*., 2016) was employed to predict the maximum biomass growth rate. The model was built on the maximum specific growth rate as a function of light intensity and water temperature. Self‐shading caused light attenuation is calculated based on biomass density. The model was validated by healthy algae pond culture grown under simulated outdoor conditions (Huesemann *et al*., 2016). Therefore, it was expected that observed growth rates of healthy cultures could match or exceed those that the model predicted.

In the four outdoor experiments, samples were taken twice daily for AFDW measurements, in the morning and in the evening. The observed growth rate was calculated via,

(1) $$\mu_{\text{obs}}=\frac{\text{Ln}\left(\frac{{\text{AFDW}}_{e}}{{\text{AFDW}}_{m}}\right)}{t_{e}-t_{m}}$$

where *μ*_obs_ is observed growth rate in day^−1^, AFDW_m_ is the morning AFDW in g l^−1^, AFDW_e_ is the evening AFDW in g l^−1^, *t*_m_ is the morning sampling time, and *t*_e_ is the evening sampling time.

The model simulates the growth during the day by setting the initial AFDW to AFDW_m_ at morning sampling time. Using the measured solar radiation and water temperature as inputs, by incorporating growth limiting factors (Gao *et al*., 2018; Khawam *et al*., 2019), the model yields the AFDW for the evening sampling time, AFDW_e_ʹ. The modelled growth rate, *μ*_model_, is calculated following Equation (1) by replacing AFDW_e_ with AFDW_e_ʹ_._

The culture Health Index (HI) is calculated via,

(2) $$\text{Health Index}\;\left(\text{HI}\right)=-\left|\frac{\mu_{\text{obs}}-\mu_{\text{model}}}{\mu_{\text{obs}}}\right|$$

Because the model only predicts growth under healthy growth conditions whereas outdoor culture is exposed to various stresses, for example, contamination, the predicted growth rate is sometimes larger than the observed growth rate (*μ*_obs_ < *μ*_model_). To facilitate interpretation and visualization, the negative sign of each HI value was taken for plotting and regression against log ratios of 16S rRNA gene abundances such that greater values reflect a higher degree of culture health.

### Total DNA isolation

Total genomic DNA was isolated from algal samples using a modified cetyltrimethylammonium bromide (CTAB) method according to Phillips *et al*. (2001). Samples were collected from turbulent sections of outdoor reactors into 50 ml tubes, and the biomass was collected by centrifugation at 4500 × *g* for 5 min. The supernatant was discarded, and 20 mg of 1.4 mm stainless steel beads was added to the pellet, with 1 ml CTAB buffer containing 20 μl β‐mercaptoethanol. The cells were disrupted using a Mini‐Beadbeater‐96 (Bio spec. Products, Bartlesville, OK). The supernatant was transferred to a sterile microfuge tube, to which an equal volume of chloroform: isoamyl alcohol (24:1) was added, and the contents were mixed by inverting each tube ten times. The emulsion was broken by centrifugation at 7500 × *g* for 10 min, and the supernatant was transferred to a microfuge tube. The supernatant was transferred to a sterile microfuge tube, and total nucleic acids were precipitated by the addition of 2/3 vol of cold isopropanol. After an overnight (~16 h) incubation at −20°C, the nucleic acids were collected by microcentrifugation, at 7500 × *g* for 10 min. The pellet was washed with 70% ethanol, air‐dried and dissolved in 20 μl Tris‐HCl buffer (TE), pH 7.2.

### 16S rRNA gene high‐throughput sequencing

The V4 region of the 16S rRNA gene (250 bp) was amplified from the purified DNA preparations by PCR using 515F and 806R primers (Parada *et al*., 2016), each harbouring a barcode such that each product received a unique tag corresponding to the sample (Caporaso *et al*., 2012) and 5 PRIME HotMasterMix (5 PRIME, Hamburg, Germany). Amplicons were quantified using Picogreen (Invitrogen, Carlsbad, CA, USA), and 240 ng of PCR amplicons from each sample was pooled. 500 µl of pooled library material was run on an electrophoresis gel to remove primer dimers and non‐specific PCR amplicons. An ~ 400 bp band was excised from the gel using the UltraClean DNA Purification and UltraClean PCR Clean‐Up Kits (Qiagen, Hilden, Germany). The pooled library was analysed by quantitative PCR amplification using the KAPA Library Quantification Kit (KAPA Biosystems, Wilmington, MA, USA), and 5% of control PhiX library (Illumina) was added to improve sequencing quality due to the low complexity of 16S rRNA gene amplicon libraries (Kozich *et al*., 2013). Paired‐end sequencing was carried out using 7pM of the pooled libraries as input on an Illumina MiSeq (Illumina, San Diego, CA, USA).

### 16S rRNA gene amplicon sequence analysis

The raw 16S rRNA gene sequence reads were first demultiplexed by assigning each read to the sample of origin according to their unique primer barcode sequences using the ‘demux’ plugin of QIIME2 (Bolyen *et al*., 2019). Forward and reverse reads were truncated at 150 bp to remove low‐quality reads, and Illumina base calling errors were resolved with the DADA2 algorithm as implemented by the ‘denoise‐paired’ method in QIIME2 (Callahan *et al*., 2016). These steps produced amplicon sequence variants (ASVs) by collapsing high‐quality reads with 100% sequence identity into groups, conceptually analogous to operational taxonomic units (OTUs). Each ASV was assigned a taxonomy by a naïve Bayes machine learning algorithm that was trained on the SILVA database (https://www.arb‐silva.de/, version 132) implemented in the q2‐feature‐classifier plugin for QIIME2 (Bokulich *et al*., 2018). Subsequent analysis of the ASVs was restricted to sequences presumed to represent bacteria associated with the algal cultures, by removing (filtering) those identified as chloroplast or mitochondria sequences. The bacterial ASVs were aligned using the MAFFT algorithm (Katoh and Standley, 2013), and a phylogenetic tree was reconstructed using fasttree 2, with a maximum‐likelihood nearest‐neighbour interchange calculation (Price *et al*., 2010). The alpha diversity of the bacterial ASVs identified in each sample was calculated based on frequency, occurrence and phylogenetic distances using the Faith’s phylogenetic diversity (PD) metric (Faith, 1992).

### Longitudinal analysis of phycosphere development in baseline experiments

The baseline samples (*n* = 165) were grouped by similarity of membership in the phycosphere using unsupervised clustering and fit to Dirichlet multinomial models (DMM) and the ASV frequency per sample as input values. The DMMs were calculated using the Dirichlet Multinomial package for R (Morgan, 2020), using a previously described method (Holmes *et al*., 2012). The fitness of the model was evaluated by calculating the Laplace approximation on a range of possible Dirichlet components or number of sample clusters. The samples were ordered by time in culture and assembled into a matrix of DMM cluster membership changes between each day. Transition frequencies were visualized by the adjacency method of the igraph R library (https://www.rdocumentation.org/packages/igraph).

### Statistical analyses of relationships between phycosphere content and variables over two growing seasons

A table of ASV frequency for each sample (*n* = 575) and eight variables from the associated metadata file (Table S6), including benzalkonium chloride treatment (BAC), days after inoculation (DAI), ash‐free dry weight (AFDW), dissolved oxygen (DO), biomass productivity, temperature, precipitation, solar radiation and wind speed were used as inputs to build a multinomial regression model based on the Multinomial function in Tensorflow (https://github.com/biocore/songbird/tree/9ed4ede40d8bd8188e93b71d1300c5a1a0a19320; Morton *et al*., 2019). The result of the multinomial model was a weighted differential value assigned to each ASV for each of the eight variables. The ASVs with *Z*‐scores greater than 3 (i.e. three standard deviations from the mean) were identified as being significantly associated with each of the variables and were summarized by bar plots of their membership to bacterial orders. The relationships between selected bacterial taxa and continuous variables were further corroborated by drawing scatterplots between the log ratios of sequence counts and the variable of interest. Linear relationships were tested by Pearson correlation calculation in base R after testing assumptions for normal distribution and homoscedasticity of residuals.

Pairwise distance metrics were calculated between all samples by the Jaccard, Bray–Curtis, unweighted unifrac and weighted unifrac methods (Faith *et al*., 1987; Chang *et al*., 2011), and principal analyses of the resulting matrices were constructed in QIIME2 (Halko *et al*., 2011) and then visualized using emperor (Vázquez‐Baeza *et al*., 2013). The effect of BAC treatment on bacterial phycospheres was investigated by comparing the ASV content of samples (*n* = 476) using the PERMANOVA method as implemented in QIIME2 (Anderson, 2001). The ASVs with significantly different relative abundances between untreated and BAC‐treated samples were identified by conducting analysis of composition of microbiomes (ANCOM) with a centred log ratio transformation (Mandal *et al*., 2015).

## Conflict of interest

The authors declare they have no conflicts of interest.

## Supporting information


**Fig. S1.** Baseline phycosphere diversity is positively correlated with duration in outdoor reactors. 16S rRNA gene sequence diversity data plotted by days after inoculation in outdoor reactor. Lines indicate linear regression with 95% confidence intervals in grey. The R fit values and p values were determined by Pearson correlation analyses.

**Fig. S2.** Optimal Dirichlet Multinomial Model cluster number for baseline data set. Goodness of fit measured by Laplace approximation as a function of the number of Dirichlet components. Lower Laplace values indicate increased model fitness to the data set.

**Fig. S3.** Monthly distribution of survey data set sample collections.

**Fig. S4.** Correlation between sequence variants and variables measured in the outdoor RAFT reactor during cultivation of *Chlorella sorokiniana*. Boxplots indicate the distribution of differential constants assigned to each 16S ribosomal RNA gene sequence variant, based on the multinomial regression against nine variables. Values outside the ± 1.5 interquartile range were designated as outliers and denoted as points outside the whiskers for each variable.

**Fig. S5.** Comparisons of algal growth metrics. Scatter plots of ash free dry weight (AFDW), biomass productivity (biomass_prod), daily average dissolved oxygen (DO), and days after inoculation (DAI) were drawn based on measurements taken during phycosphere sampling time points. Lines represent Pearson correlation with shaded areas indicating the 95% confidence intervals.

**Fig. S6.** The bacterial orders identified as most highly correlated with biomass productivity of the algal cultures. Counts of sequence variants with multinomial regression differential values for biomass productivity are indicated by a z score of greater than three. The sequence variants are grouped by the assigned taxonomy at the order‐level, and bars are color‐coded based on the direction of the correlation.

**Fig. S7.** Log ratio relationship of select bacterial orders to biomass productivity. The log ratios of the relative abundance of (a) Betaproteobacteriales ASVs and (b) Rhizobiales to Vampirovibrionales for each sample are plotted by their corresponding biomass productivity measure. Lines and grey areas represent linear regression and 95% confidence intervals, respectively, and R fitness and p significance values, based on the Pearson’s correlation test.

**Fig. S8.** Bacterial orders most highly correlated with the duration algal cultivation in the outdoor RAFT reactors. Counts of sequence variants with multinomial regression differential values for days after inoculation are indicated by a z score of greater than three. The sequence variants are grouped by their assigned taxonomy at the order‐level, and bars are color‐coded by the direction of the correlation.

**Fig. S9.** Log ratio relationships of selected bacterial taxa to algal biomass productivity. The log ratios of the relative abundance of (a) *Rhizobium sp*. (accession no. JX255399), (b) Vampirovibrionales, (c) Burholderiaceae ASV 21c587e98537024a404d6f041c0fb693, (d) Rhodobacterales, (e) Burkholderiaceae, and (f) Cytophagales to Sphingobacteriaceae for each sample are plotted by their corresponding biomass productivity measure. Lines and grey areas represent linear regression and 95% confidence intervals, respectively, and R fitness and *P* significance values, based on the Pearson’s correlation test.

**Fig. S10.** Comparisons of simulated and observed algal growth rates during RAFT baseline experiments. Observed and simulated algal culture growth rates for all four runs are shown (a) with correlation curves drawn based on loess approximation and 95% confidence intervals indicated by shaded areas. Boxplots (b) indicating the distributions of calculated absolute relative error of growth rates (mu) are displayed by days after inoculation, and the relationship between observed growth rate and calculated absolute relative error of growth rate (c) for the four duplicate runs.

**Fig. S11.** Bacterial orders most highly correlated with algal Health Index (HI). Counts of sequence variants with multinomial regression differential values for days after inoculation that have a z score greater than three. The sequence variants are grouped by their assigned taxonomies at the order level, and bars are color coded by the direction of the correlation.

**Fig. S12.** Comparisons of putative algal culture performance metrics for identification of bacterial interactions. The log ratios of the relative abundance of (a–c) Allorhizobium‐Neorhizobium‐Pararhizobium‐Rhizobium (ANPR) and (d–f) *Vampirovibrio* sp. to Methylophilus for each sample are plotted by their corresponding algal HI measure, biomass productivity, and ash free dry weight (AFDW). Lines and grey areas represent linear regression and 95% confidence intervals respectively, along with the R fitness and p significance values based on a Pearson correlation test.

**Fig. S13.** Distributions of (A) biomass productivity (*P* = 0.51) and (B) temperature (*P* = 2.32e−10) measurements for samples collected from cultures treated (blue) or untreated (orange) with benzalkonium chloride.

**Table S1.** RAFT baseline experiments continuous data summary.

**Table S2.** Multinomial model differentials for all amplicon sequence variants and their taxonomic assignments.
**Table S3** **.** Benzalkonium beta group significance PERMANOVA results.
**Table S4** **.** Most significant sequence variants to biomass productivity according to multinomial model.
**Table S5** **.** Comparison of bacterial phyla significantly changed by benzalkonium chloride treatment to total amplicon sequence variant taxonomic assignments.

**Table S6.** RAFT 16S rRNA gene sample metadata table.

## Data Availability

These sequence data have been submitted to the National Center for Biotechnology Information Sequence Read Archive under accession number PRJNA597200.

## References

[mbt213591-bib-0001] Amavizca, E. , Bashan, Y. , Ryu, C.‐M. , Farag, M.A. , Bebout, B.M. , and de‐Bashan, L.E. (2017) Enhanced performance of the microalga *Chlorella sorokiniana* remotely induced by the plant growth‐promoting bacteria *Azospirillum brasilense* and *Bacillus pumilus* . Sci Rep 7: 41310. 28145473 10.1038/srep41310 PMC5286510

[mbt213591-bib-0002] Anderson, M.J. (2001) A new method for non‐parametric multivariate analysis of variance. Austral Ecol 26: 32–46.

[mbt213591-bib-0003] Bashan, Y. , Lopez, B.R. , Huss, V.A.R. , Amavizca, E. , and de‐Bashan, L.E. (2016) *Chlorella sorokiniana* (formerly *C. vulgaris*) UTEX 2714, a non‐thermotolerant microalga useful for biotechnological applications and as a reference strain. J Appl Phycol 28: 113–121.

[mbt213591-bib-0004] Bell, W. , and Mitchell, R. (1972) Chemotactic and growth responses of marine bacteria to algal extracellular products. Biol Bull 143: 265–277.

[mbt213591-bib-0005] Berendsen, R.L. , Pieterse, C.M.J. , and Bakker, P.A.H.M. (2012) The rhizosphere microbiome and plant health. Trends Plant Sci 17: 478–486. 22564542 10.1016/j.tplants.2012.04.001

[mbt213591-bib-0006] Blanc, G. , Duncan, G. , Agarkova, I. , Borodovsky, M. , Gurnon, J. , Kuo, A. , *et al* (2010) The *Chlorella variabilis* NC64A genome reveals adaptation to photosymbiosis, coevolution with viruses, and cryptic sex. Plant Cell 22: 2943–2955. 20852019 10.1105/tpc.110.076406 PMC2965543

[mbt213591-bib-0007] Bokulich, N.A. , Kaehler, B.D. , Rideout, J.R. , Dillon, M. , Bolyen, E. , Knight, R. , *et al* (2018) Optimizing taxonomic classification of marker‐gene amplicon sequences with QIIME 2’s q2‐feature‐classifier plugin. Microbiome 6: 90. 29773078 10.1186/s40168-018-0470-z PMC5956843

[mbt213591-bib-0008] Bolyen, E. , Rideout, J.R. , Dillon, M.R. , Bokulich, N.A. , Abnet, C.C. , Al‐Ghalith, G.A. , *et al* (2019) Reproducible, interactive, scalable and extensible microbiome data science using QIIME 2. Nat Biotechnol 37: 852–857. 31341288 10.1038/s41587-019-0209-9 PMC7015180

[mbt213591-bib-0009] Callahan, B.J. , McMurdie, P.J. , Rosen, M.J. , Han, A.W. , Johnson, A.J.A. , and Holmes, S.P. (2016) DADA2: High‐resolution sample inference from Illumina amplicon data. Nat Methods 13: 581–583. 27214047 10.1038/nmeth.3869 PMC4927377

[mbt213591-bib-0010] Callahan, B.J. , McMurdie, P.J. , and Holmes, S.P. (2017) Exact sequence variants should replace operational taxonomic units in marker‐gene data analysis. ISME J 11: 2639–2643. 28731476 10.1038/ismej.2017.119 PMC5702726

[mbt213591-bib-0011] Caporaso, J.G. , Lauber, C.L. , Walters, W.A. , Berg‐Lyons, D. , Huntley, J. , Fierer, N. , *et al* (2012) Ultra‐high‐throughput microbial community analysis on the Illumina HiSeq and MiSeq platforms. ISME J 6: 1621–1624. 22402401 10.1038/ismej.2012.8 PMC3400413

[mbt213591-bib-0012] Carney, L.T. , and Lane, T.W. (2014) Parasites in algae mass culture. Aquat Microbiol 5: 278. 10.3389/fmicb.2014.00278 PMC4047527 24936200

[mbt213591-bib-0013] Carney, L.T. , Reinsch, S.S. , Lane, P.D. , Solberg, O.D. , Jansen, L.S. , Williams, K.P. , *et al* (2014) Microbiome analysis of a microalgal mass culture growing in municipal wastewater in a prototype OMEGA photobioreactor. Algal Res 4: 52–61.

[mbt213591-bib-0014] Chang, Q. , Luan, Y. , and Sun, F. (2011) Variance adjusted weighted UniFrac: a powerful beta diversity measure for comparing communities based on phylogeny. BMC Bioinformatics 12: 118. 21518444 10.1186/1471-2105-12-118 PMC3108311

[mbt213591-bib-0015] Coder, D.M. , and Goff, L.J. (1986) The host range of the chlorellavorous bacterium (“*Vampirovibrio chlorellavorus*”). J Phycol 22: 543–546.

[mbt213591-bib-0016] Cole, J.J. (1982) Interactions between bacteria and algae in aquatic ecosystems. Annu Rev Ecol Syst 13: 291–314.

[mbt213591-bib-0017] Crowe, B. , Attalah, S. , Agrawal, S. , Waller, P. , Ryan, R. , Van Wagenen, J. , *et al* (2012) A comparison of *Nannochloropsis salina* growth performance in two outdoor pond designs: conventional raceways versus the arid pond with superior temperature management. Int J Chem Eng 2012: 1–9.

[mbt213591-bib-0018] De‐Bashan, L.E. , Antoun, H. , and Bashan, Y. (2008) Involvement of Indole‐3‐Acetic acid produced by the growth‐promoting bacterium *Azospirillum spp*. in promoting growth of *Chlorella Vulgaris* . J Phycol 44: 938–947. 27041612 10.1111/j.1529-8817.2008.00533.x

[mbt213591-bib-0019] Edgar, R.C. (2018) Updating the 97% identity threshold for 16S ribosomal RNA OTUs. Bioinformatics 34: 2371–2375. 29506021 10.1093/bioinformatics/bty113

[mbt213591-bib-0020] Faith, D.P. (1992) Conservation evaluation and phylogenetic diversity. Biol Conserv 61: 1–10.

[mbt213591-bib-0021] Faith, D.P. , Minchin, P.R. , and Belbin, L. (1987) Compositional dissimilarity as a robust measure of ecological distance. Vegetatio 69: 57–68.

[mbt213591-bib-0022] Gachon, C.M.M. , Sime‐Ngando, T. , Strittmatter, M. , Chambouvet, A. , and Kim, G.H. (2010) Algal diseases: spotlight on a black box. Trends Plant Sci 15: 633–640. 20833575 10.1016/j.tplants.2010.08.005

[mbt213591-bib-0023] Ganuza, E. , Sellers, C.E. , Bennett, B.W. , Lyons, E.M. , and Carney, L.T. (2016) A Novel treatment protects chlorella at commercial scale from the predatory Bacterium Vampirovibrio chlorellavorus. Front Microbiol 7: 848. 27379027 10.3389/fmicb.2016.00848 PMC4913114

[mbt213591-bib-0024] Gao, S. , Waller, P. , Khawam, G. , Attalah, S. , Huesemann, M. , and Ogden, K. (2018) Incorporation of salinity, nitrogen, and shading stress factors into the Huesemann Algae Biomass Growth model. Algal Res 35: 462–470.

[mbt213591-bib-0025] Gromov, B.V. , and Mamkaeva, K.A. (1972) Electron microscopic study of parasitism by *Bdellovibrio chlorellavorus* bacteria on cells of the green alga *Chlorella vulgaris* . Tsitologiia 14: 256–260. 5011884

[mbt213591-bib-0026] Halko, N. , Martinsson, P. , Shkolnisky, Y. , and Tygert, M. (2011) An Algorithm for the principal component analysis of large data sets. SIAM J Sci Comput 33: 2580–2594.

[mbt213591-bib-0027] Hernandez, J.‐P. , de‐Bashan, L.E. , Rodriguez, D.J. , Rodriguez, Y. , and Bashan, Y. (2009) Growth promotion of the freshwater microalga *Chlorella vulgaris* by the nitrogen‐fixing, plant growth‐promoting bacterium *Bacillus pumilus* from arid zone soils. Eur J Soil Biol 45: 88–93.

[mbt213591-bib-0028] Holmes, I. , Harris, K. , and Quince, C. (2012) Dirichlet multinomial mixtures: generative models for microbial metagenomics. PLoS ONE 7: e30126. 22319561 10.1371/journal.pone.0030126 PMC3272020

[mbt213591-bib-0029] Hu, Q. , Sommerfeld, M. , Jarvis, E. , Ghirardi, M. , Posewitz, M. , Seibert, M. , and Darzins, A. (2008) Microalgal triacylglycerols as feedstocks for biofuel production: perspectives and advances. Plant J 54: 621–639. 18476868 10.1111/j.1365-313X.2008.03492.x

[mbt213591-bib-0030] Huesemann, M.H. , Van Wagenen, J. , Miller, T. , Chavis, A. , Hobbs, S. , and Crowe, B. (2013) A screening model to predict microalgae biomass growth in photobioreactors and raceway ponds. Biotechnol Bioeng 110: 1583–1594. 23280255 10.1002/bit.24814

[mbt213591-bib-0031] Huesemann, M. , Crowe, B. , Waller, P. , Chavis, A. , Hobbs, S. , Edmundson, S. , and Wigmosta, M. (2016) A validated model to predict microalgae growth in outdoor pond cultures subjected to fluctuating light intensities and water temperatures. Algal Res 13: 195–206.

[mbt213591-bib-0032] Imai, I. , Ishida, Y. , and Hata, Y. (1993) Killing of marine phytoplankton by a gliding bacterium *Cytophaga sp*., isolated from the coastal sea of Japan. Mar Biol 116: 527–532.

[mbt213591-bib-0033] Jorquera, O. , Kiperstok, A. , Sales, E.A. , Embiruçu, M. , and Ghirardi, M.L. (2010) Comparative energy life‐cycle analyses of microalgal biomass production in open ponds and photobioreactors. Bioresour Technol 101: 1406–1413. 19800784 10.1016/j.biortech.2009.09.038

[mbt213591-bib-0034] Katoh, K. , and Standley, D.M. (2013) MAFFT Multiple Sequence Alignment Software Version 7: improvements in performance and usability. Mol Biol Evol 30: 772–780. 23329690 10.1093/molbev/mst010 PMC3603318

[mbt213591-bib-0035] Khawam, G. , Waller, P. , Gao, S. , Edmundson, S. , Huesemann, M. , Attalah, S. , and Ogden, K.L. (2019) Simulation of shading and algal growth in experimental raceways. Algal Res 41: 101575.

[mbt213591-bib-0036] Kim, B.‐H. , Ramanan, R. , Cho, D.‐H. , Oh, H.‐M. , and Kim, H.‐S. (2014) Role of Rhizobium, a plant growth promoting bacterium, in enhancing algal biomass through mutualistic interaction. Biomass Bioenergy 69: 95–105.

[mbt213591-bib-0037] Kovacevic, J. , Ziegler, J. , Wałecka‐Zacharska, E. , Reimer, A. , Kitts, D.D. , and Gilmour, M.W. (2016) Tolerance of *Listeria monocytogenes* to quaternary ammonium sanitizers is mediated by a novel efflux pump encoded by emrE. Appl Environ Microbiol 82: 939–953. 26590290 10.1128/AEM.03741-15 PMC4725271

[mbt213591-bib-0038] Kozich, J.J. , Westcott, S.L. , Baxter, N.T. , Highlander, S.K. , and Schloss, P.D. (2013) Development of a dual‐index sequencing strategy and curation pipeline for analyzing amplicon sequence data on the MiSeq Illumina Sequencing Platform. Appl Environ Microbiol 79: 5112–5120. 23793624 10.1128/AEM.01043-13 PMC3753973

[mbt213591-bib-0039] Krauss, R.W. , and Thomas, W.H. (1954) The Growth and inorganic nutrition of *Scenedesmus obliquus* in mass culture. Plant Physiol 29: 205–214. 16654644 10.1104/pp.29.3.205 PMC540499

[mbt213591-bib-0040] Lammers, P.J. , Huesemann, M. , Boeing, W. , Anderson, D.B. , Arnold, R.G. , Bai, X. , *et al* (2017) Review of the cultivation program within the National Alliance for Advanced Biofuels and Bioproducts. Algal Res 22: 166–186.

[mbt213591-bib-0041] Lugtenberg, B. , and Kamilova, F. (2009) Plant‐growth‐promoting Rhizobacteria. Annu Rev Microbiol 63: 541–556. 19575558 10.1146/annurev.micro.62.081307.162918

[mbt213591-bib-0042] Mandal, S. , Treuren, W.V. , White, R.A. , Eggesbø, M. , Knight, R. , and Peddada, S.D. (2015) Analysis of composition of microbiomes: a novel method for studying microbial composition. Microb Ecol Health Dis 26: 27663. 26028277 10.3402/mehd.v26.27663 PMC4450248

[mbt213591-bib-0043] Mayali, X. , and Azam, F. (2004) Algicidal bacteria in the sea and their impact on algal blooms. J Eukaryot Microbiol 51: 139–144. 15134248 10.1111/j.1550-7408.2004.tb00538.x

[mbt213591-bib-0078] Metting F.B. (1996) Biodiversity and application of microalgae. Journal of Industrial Microbiology & Biotechnology, 17(5–6), 477–489. 10.1007/bf01574779.

[mbt213591-bib-0044] Morgan, M. (2020) DirichletMultinomial: Dirichlet‐Multinomial Mixture Model Machine Learning for Microbiome Data. R package, version 1.30.0.

[mbt213591-bib-0045] Morton, J.T. , Marotz, C. , Washburne, A. , Silverman, J. , Zaramela, L.S. , Edlund, A. , *et al* (2019) Establishing microbial composition measurement standards with reference frames. Nat Commun 10: 2719. 31222023 10.1038/s41467-019-10656-5 PMC6586903

[mbt213591-bib-0046] Myers, J. , Phillips, J.N. , and Graham, J.‐R. (1951) On the mass culture of algae. Plant Physiol 26: 539–548. 16654390 10.1104/pp.26.3.539 PMC437522

[mbt213591-bib-0047] Nadeem, S.M. , Ahmad, M. , Zahir, Z.A. , Javaid, A. , and Ashraf, M. (2014) The role of mycorrhizae and plant growth promoting rhizobacteria (PGPR) in improving crop productivity under stressful environments. Biotechnol Adv 32: 429–448. 24380797 10.1016/j.biotechadv.2013.12.005

[mbt213591-bib-0048] Neofotis, P. , Huang, A. , Sury, K. , Chang, W. , Joseph, F. , Gabr, A. , *et al* (2016) Characterization and classification of highly productive microalgae strains discovered for biofuel and bioproduct generation. Algal Res 15: 164–178.

[mbt213591-bib-0049] Ogden, K. , Anderson, D. , Simpson, S. , Van Voorheis, W. , Brown, J. , Huesemann, M. , *et al* (2019) Regional Algal Feedstock Testebed (RAFT). Final Report, Univ. of Arizona, Tucson, AZ (United States).

[mbt213591-bib-0050] Parada, A.E. , Needham, D.M. , and Fuhrman, J.A. (2016) Every base matters: assessing small subunit rRNA primers for marine microbiomes with mock communities, time series and global field samples. Environ Microbiol 18: 1403–1414. 26271760 10.1111/1462-2920.13023

[mbt213591-bib-0051] Park, S.‐H. , Steichen, S.A. , Li, X. , Ogden, K. , and Brown, J.K. (2018) Association of *Vampirovibrio chlorellavorus* with decline and death of *Chlorella sorokiniana* in outdoor reactors. J Appl Phycol 31: 1131–1142.

[mbt213591-bib-0052] Pedroni, P.M. , Lamenti, G. , Prosperi, G. , Ritorto, L. , Scolla, G. , Capuano, F. , and Valdiserri, M. (2004) EniTecnologie R&D project on microalgae biofixation of CO2: outdoor comparative tests of biomass productivity using flue gas CO2 from a NGCC power plant. Proc GHGT 7: 5–9.

[mbt213591-bib-0053] Phillips, N. , Smith, C.M. , and Morden, C.W. (2001) An effective DNA extraction protocol for brown algae. Phycol Res 49: 97–102.

[mbt213591-bib-0054] Price, M.N. , Dehal, P.S. , and Arkin, A.P. (2010) FastTree 2 – Approximately Maximum‐Likelihood Trees for Large Alignments. PLoS ONE 5: e9490. 20224823 10.1371/journal.pone.0009490 PMC2835736

[mbt213591-bib-0055] Rashidan, K.K. , and Bird, D.F. (2001) Role of predatory bacteria in the termination of a cyanobacterial bloom. Microb Ecol 41: 97–105. 12032614 10.1007/s002480000074

[mbt213591-bib-0056] Richmond, A. (ed.) (2004) Handbook of microalgal culture: biotechnology and applied phycology. Oxford, UK; Ames, IA: Blackwell Science.

[mbt213591-bib-0057] Richter, L.V. , Mansfeldt, C.B. , Kuan, M.M. , Cesare, A.E. , Menefee, S.T. , Richardson, R.E. , and Ahner, B.A. (2018) Altered microbiome leads to significant phenotypic and transcriptomic differences in a lipid accumulating chlorophyte. Environ Sci Technol 52: 6854–6863. 29750518 10.1021/acs.est.7b06581

[mbt213591-bib-0058] Rippka, R. , and Herdman, M. (1992) Pasteur culture collection of cyanobacterial strains in axenic culture. Cat Taxon Handb 1: 103.

[mbt213591-bib-0059] Rivas, R. , Velázquez, E. , Willems, A. , Vizcaíno, N. , Subba‐Rao, N.S. , Mateos, P.F. , *et al* (2002) A new species of Devosia that forms a unique nitrogen‐fixing root‐nodule symbiosis with the aquatic legume *Neptunia natans* (L.f.) Druce. Appl Env Microbiol 68: 5217–5222. 12406707 10.1128/AEM.68.11.5217-5222.2002 PMC129907

[mbt213591-bib-0060] Sawada, H. , Kuykendall, L.D. , and Young, J.M. (2003) Changing concepts in the systematics of bacterial nitrogen‐fixing legume symbionts. J Gen Appl Microbiol 49: 155–179. 12949698 10.2323/jgam.49.155

[mbt213591-bib-0061] Seymour, J.R. , Amin, S.A. , Raina, J.‐B. , and Stocker, R. (2017) Zooming in on the phycosphere: the ecological interface for phytoplankton–bacteria relationships. Nat Microbiol 2: 17065. 28555622 10.1038/nmicrobiol.2017.65

[mbt213591-bib-0062] Shihira, I. , and Krauss, R.W. (1965) Chlorella: physiology and taxonomy of forty‐one Isolates. College Park, MD: University of Maryland.

[mbt213591-bib-0063] Singh, A. , Nigam, P.S. , and Murphy, J.D. (2011) Renewable fuels from algae: an answer to debatable land based fuels. Bioresour Technol 102: 10–16. 20615690 10.1016/j.biortech.2010.06.032

[mbt213591-bib-0064] Soo, R.M. , Woodcroft, B.J. , Parks, D.H. , Tyson, G.W. , and Hugenholtz, P. (2015) Back from the dead; the curious tale of the predatory cyanobacterium *Vampirovibrio chlorellavorus* . PeerJ 3: e968. 26038723 10.7717/peerj.968 PMC4451040

[mbt213591-bib-0065] Steichen, S.A. , and Brown, J.K. (2019) Real‐time quantitative detection of *Vampirovibrio chlorellavorus*, an obligate bacterial pathogen of *Chlorella sorokiniana* . J Appl Phycol 31: 1117–1129.

[mbt213591-bib-0066] Stewart, E.J. (2012) Growing unculturable bacteria. J Bacteriol 194: 4151–4160. 22661685 10.1128/JB.00345-12 PMC3416243

[mbt213591-bib-0067] Stewart, C.J. , Ajami, N.J. , O'Brien, J.L. , Hutchinson, D.S. , Smith, D.P. , Wong, M.C. , *et al* (2018) Temporal development of the gut microbiome in early childhood from the TEDDY study. Nature 562: 583. 30356187 10.1038/s41586-018-0617-x PMC6415775

[mbt213591-bib-0068] Sze, M.A. , and Schloss, P.D. (2018) Leveraging existing 16S rRNA gene surveys to identify reproducible biomarkers in individuals with colorectal tumors. MBio: 9: e00630‐18. 29871916 10.1128/mBio.00630-18 PMC5989068

[mbt213591-bib-0069] Thompson, A.W. , Foster, R.A. , Krupke, A. , Carter, B.J. , Musat, N. , Vaulot, D. , *et al* (2012) Unicellular Cyanobacterium symbiotic with a single‐celled eukaryotic alga. Science 337: 1546–1550. 22997339 10.1126/science.1222700

[mbt213591-bib-0070] Thompson, L.R. , Sanders, J.G. , McDonald, D. , Amir, A. , Ladau, J. , Locey, K.J. , *et al* (2017) A communal catalogue reveals Earth’s multiscale microbial diversity. Nature 551: 457–463. 29088705 10.1038/nature24621 PMC6192678

[mbt213591-bib-0071] Vázquez‐Baeza, Y. , Pirrung, M. , Gonzalez, A. , and Knight, R. (2013) EMPeror: a tool for visualizing high‐throughput microbial community data. GigaScience 2: 16. 24280061 10.1186/2047-217X-2-16 PMC4076506

[mbt213591-bib-0072] Vu, C.H.T. , Lee, H.‐G. , Chang, Y.K. , and Oh, H.‐M. (2018) Axenic cultures for microalgal biotechnology: establishment, assessment, maintenance, and applications. Biotechnol Adv 36: 380–396. 29292155 10.1016/j.biotechadv.2017.12.018

[mbt213591-bib-0073] Waller, P. , Ryan, R. , Kacira, M. , and Li, P. (2012) The algae raceway integrated design for optimal temperature management. Biomass Bioenergy 46: 702–709.

[mbt213591-bib-0074] Wang, X. , Li, Z. , Su, J. , Tian, Y. , Ning, X. , Hong, H. , and Zheng, T. (2010) Lysis of a red‐tide causing alga, *Alexandrium tamarense*, caused by bacteria from its phycosphere. Biol Control 52: 123–130.

[mbt213591-bib-0075] Watanabe, K. , Takihana, N. , Aoyagi, H. , Hanada, S. , Watanabe, Y. , Ohmura, N. , *et al* (2005) Symbiotic association in Chlorella culture. FEMS Microbiol Ecol 51: 187–196. 16329867 10.1016/j.femsec.2004.08.004

[mbt213591-bib-0076] Wigmosta, M.S. , Coleman, A.M. , Skaggs, R.J. , Huesemann, M.H. , and Lane, L.J. (2011) National microalgae biofuel production potential and resource demand. Water Resour Res 47: W00H04.

[mbt213591-bib-0077] Woese, C.R. (1987) Bacterial evolution. Microbiol Rev 51: 221. 2439888 10.1128/mr.51.2.221-271.1987 PMC373105

