# Supplementary figures and images for: Association between algal productivity and phycosphere composition in an outdoor Chlorella sorokiniana reactor based on multiple longitudinal analyses
Source: Microb Biotechnol. 2020 May 25;13(5):1546–61. doi: 10.1111/1751-7915.13591 (PMC7415377; doi:10.1111/1751-7915.13591)

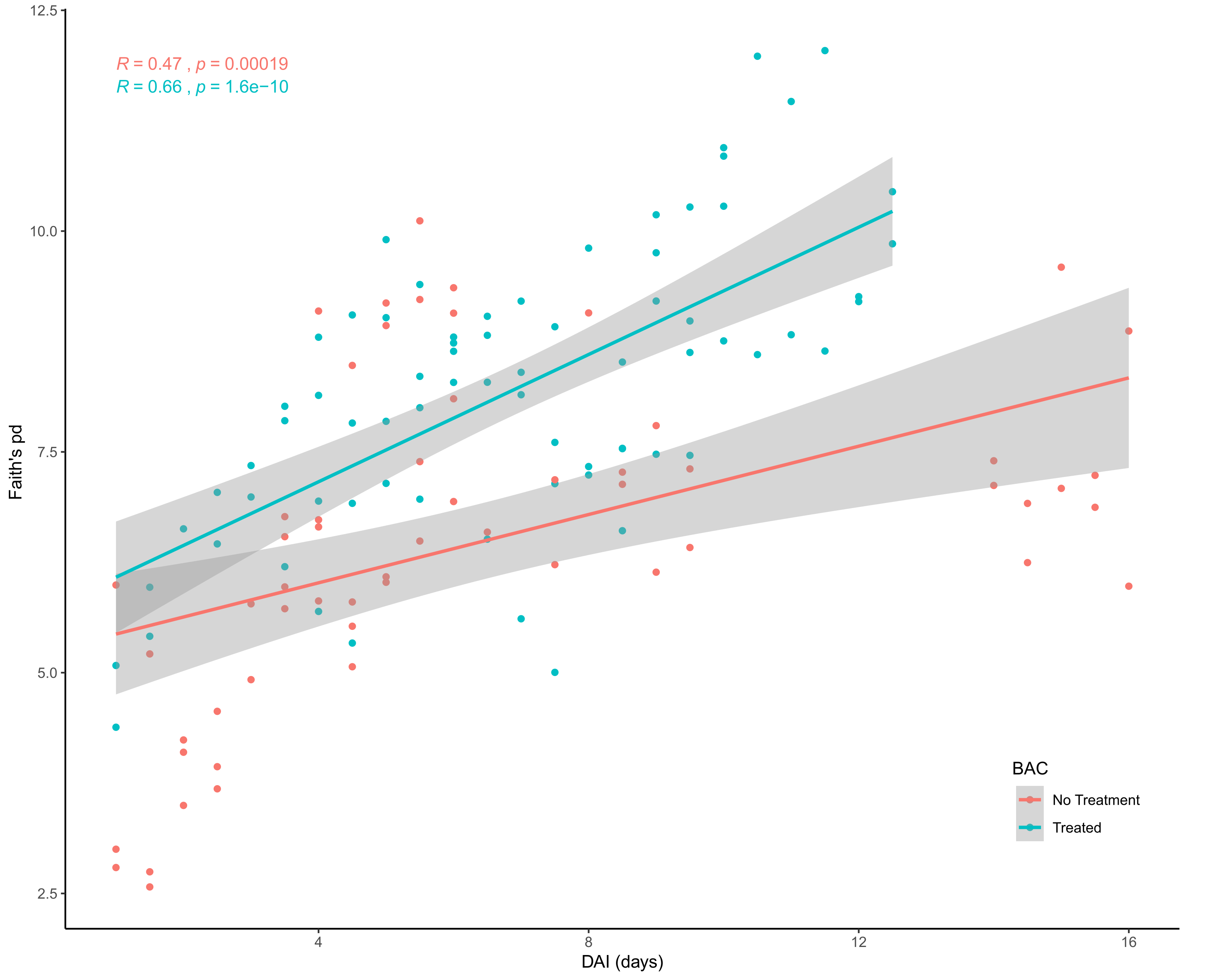

Supplement: Supplementary file 1 — Fig. S1. Baseline phycosphere diversity is positively correlated with duration in outdoor reactors. 16S rRNA gene sequence diversity data plotted by days after inoculation in outdoor reactor. Lines indicate linear regression with 95% confidence intervals in grey. The R fit values and p values were determined by Pearson correlation analyses. [file MBT2-13-1546-s001.png]

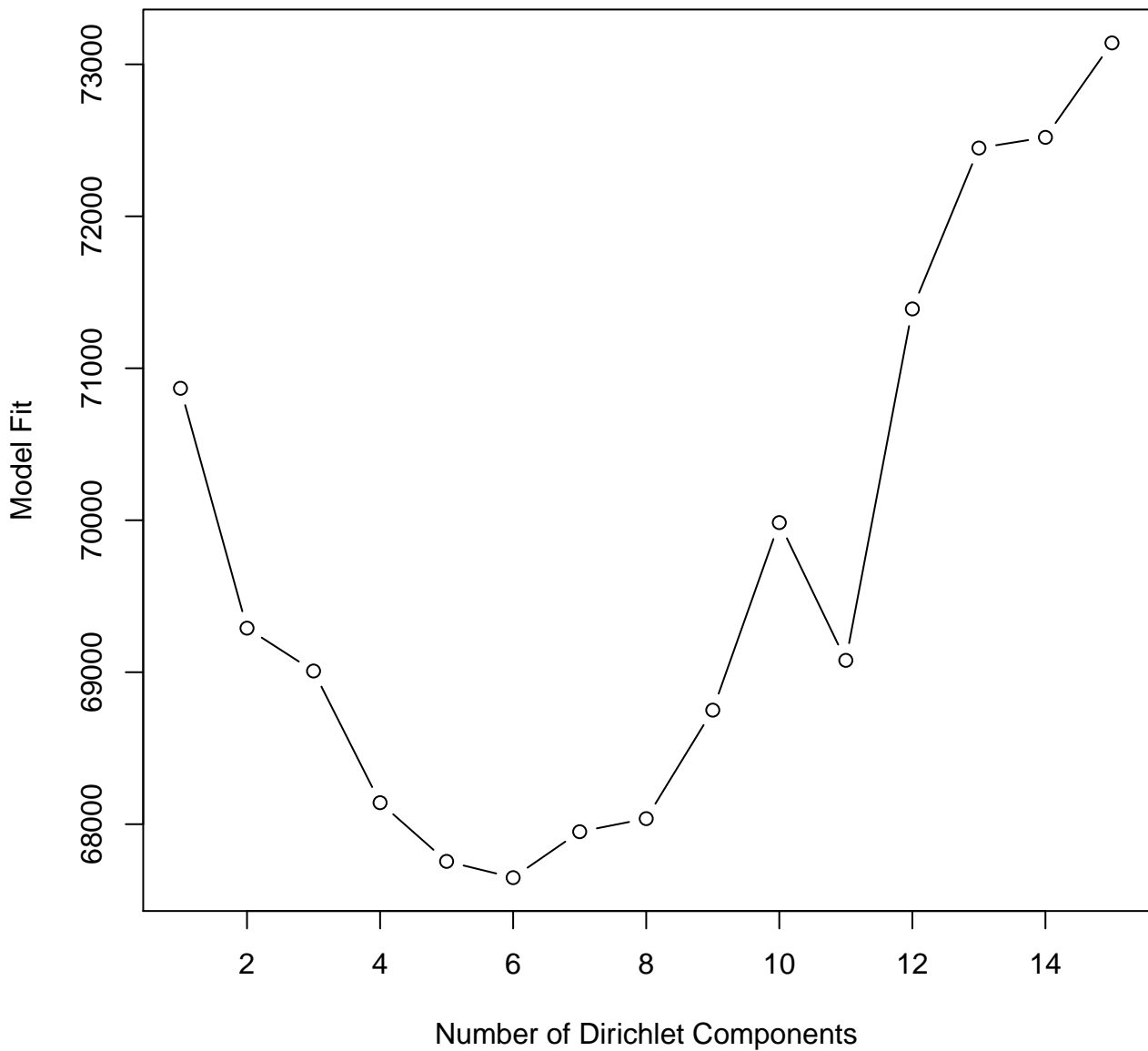

Supplement: Supplementary file 2 — Fig. S2. Optimal Dirichlet Multinomial Model cluster number for baseline data set. Goodness of fit measured by Laplace approximation as a function of the number of Dirichlet components. Lower Laplace values indicate increased model fitness to the data set. [file MBT2-13-1546-s002.pdf]

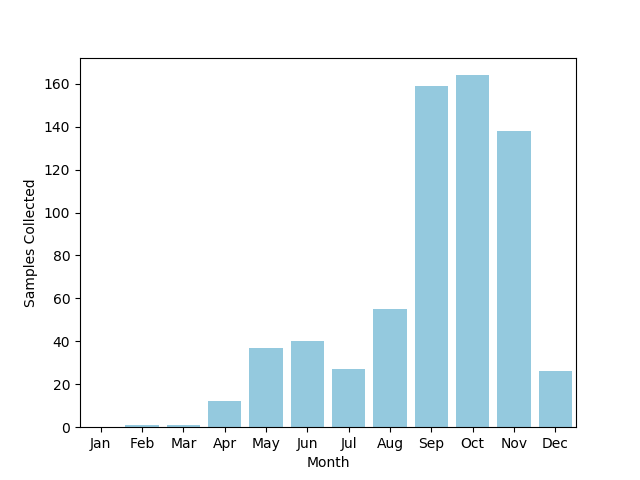

Supplement: Supplementary file 3 — Fig. S3. Monthly distribution of survey data set sample collections. [file MBT2-13-1546-s003.png]

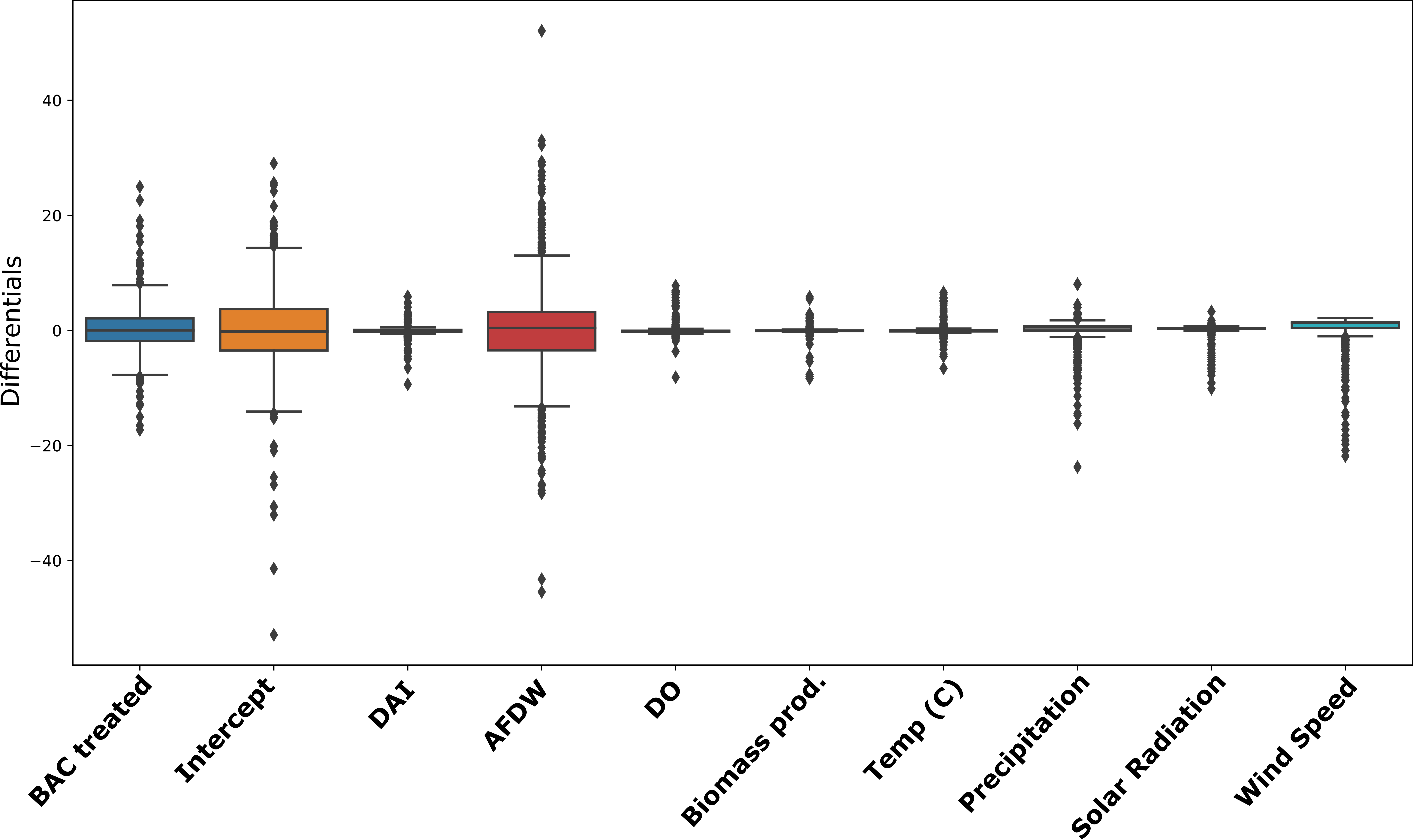

Supplement: Supplementary file 4 — Fig. S4. Correlation between sequence variants and variables measured in the outdoor RAFT reactor during cultivation of Chlorella sorokiniana. Boxplots indicate the distribution of differential constants assigned to each 16S ribosomal RNA gene sequence variant, based on the multinomial regression against nine variables. Values outside the ± 1.5 interquartile range were designated as outliers and denoted as points outside the whiskers for each variable. [file MBT2-13-1546-s004.png]

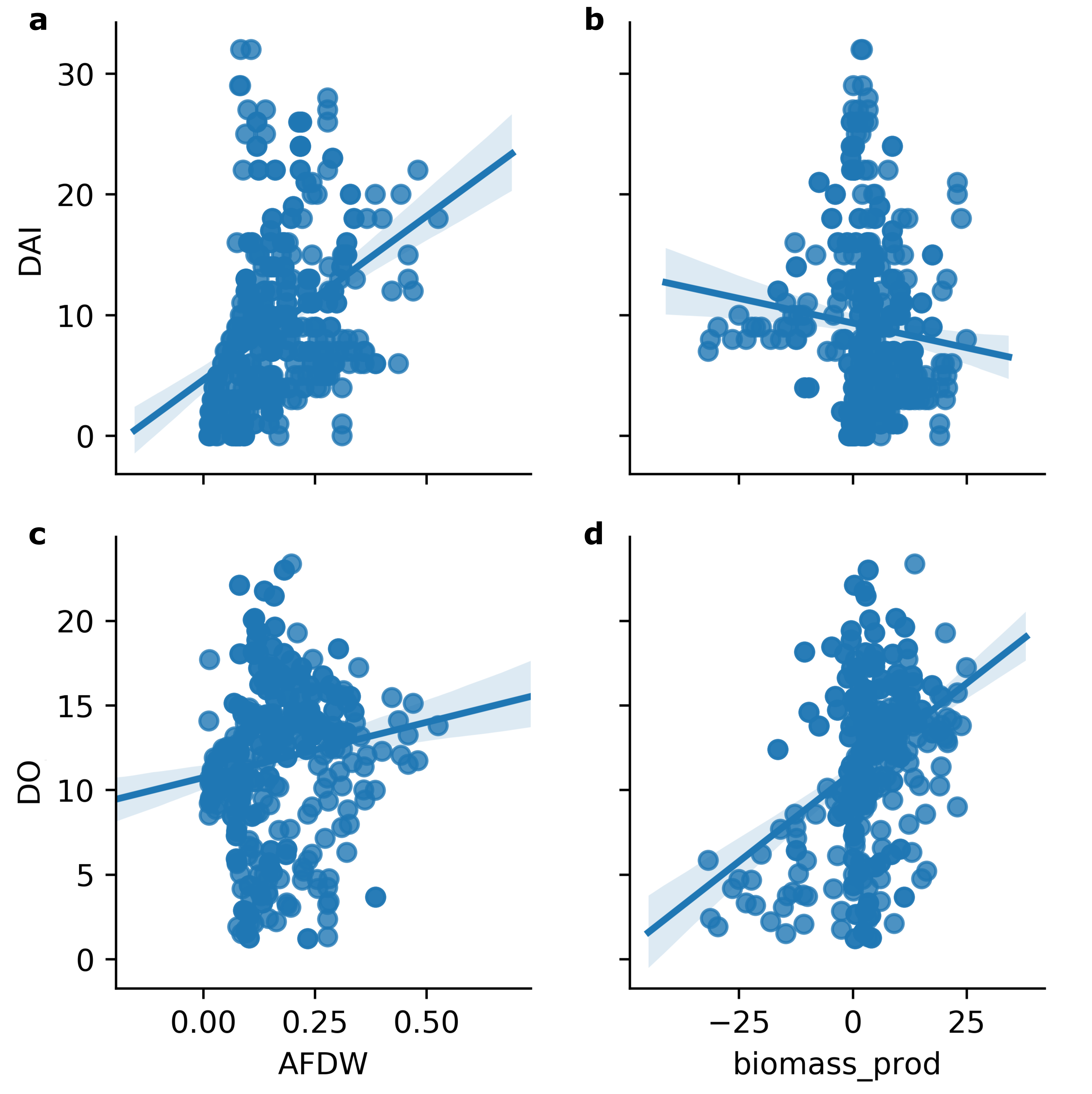

Supplement: Supplementary file 5 — Fig. S5. Comparisons of algal growth metrics. Scatter plots of ash free dry weight (AFDW), biomass productivity (biomass_prod), daily average dissolved oxygen (DO), and days after inoculation (DAI) were drawn based on measurements taken during phycosphere sampling time points. Lines represent Pearson correlation with shaded areas indicating the 95% confidence intervals. [file MBT2-13-1546-s005.png]

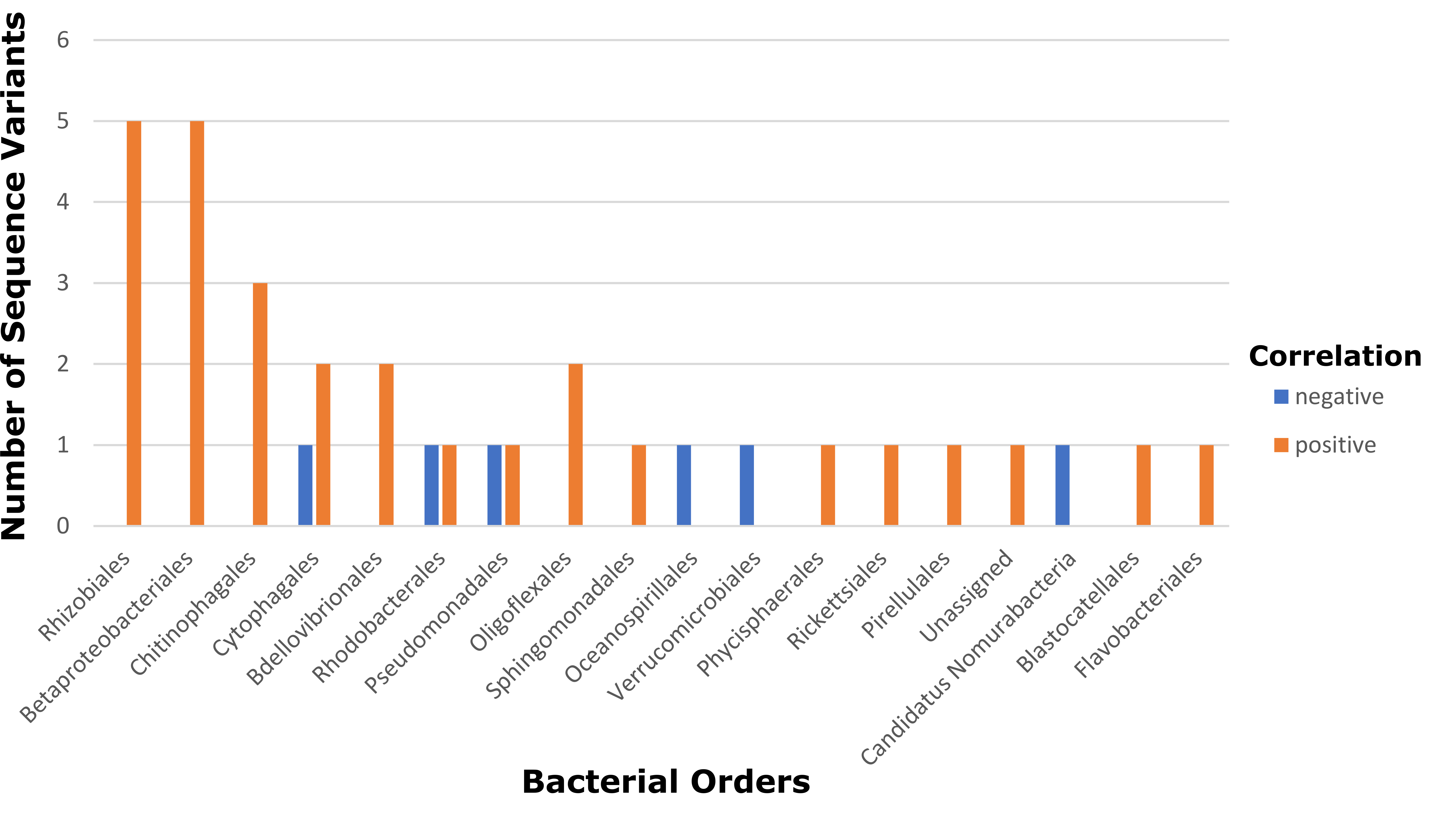

Supplement: Supplementary file 6 — Fig. S6. The bacterial orders identified as most highly correlated with biomass productivity of the algal cultures. Counts of sequence variants with multinomial regression differential values for biomass productivity are indicated by a z score of greater than three. The sequence variants are grouped by the assigned taxonomy at the order‐level, and bars are color‐coded based on the direction of the correlation. [file MBT2-13-1546-s006.png]

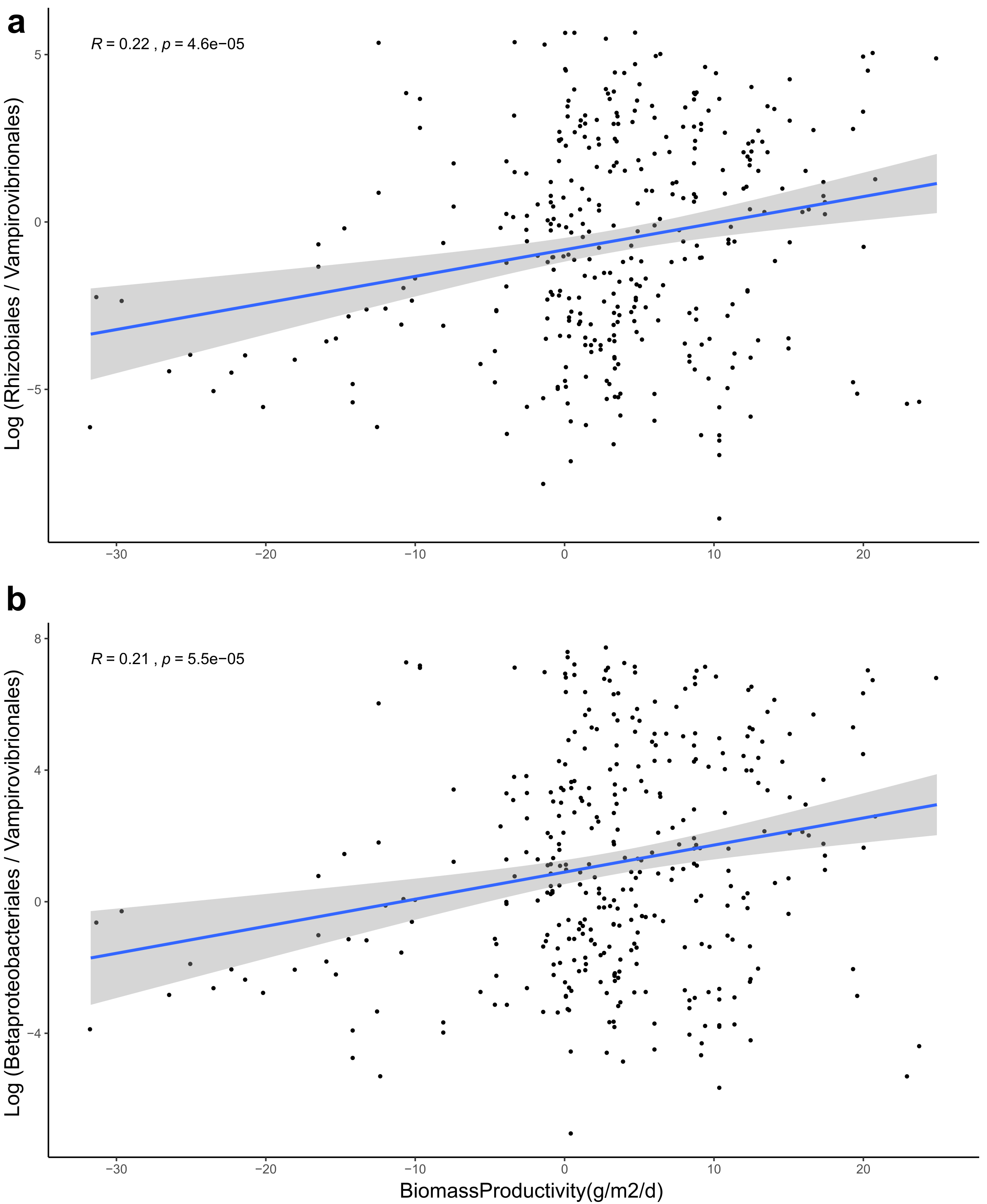

Supplement: Supplementary file 7 — Fig. S7. Log ratio relationship of select bacterial orders to biomass productivity. The log ratios of the relative abundance of (a) Betaproteobacteriales ASVs and (b) Rhizobiales to Vampirovibrionales for each sample are plotted by their corresponding biomass productivity measure. Lines and grey areas represent linear regression and 95% confidence intervals, respectively, and R fitness and p significance values, based on the Pearson’s correlation test. [file MBT2-13-1546-s007.png]

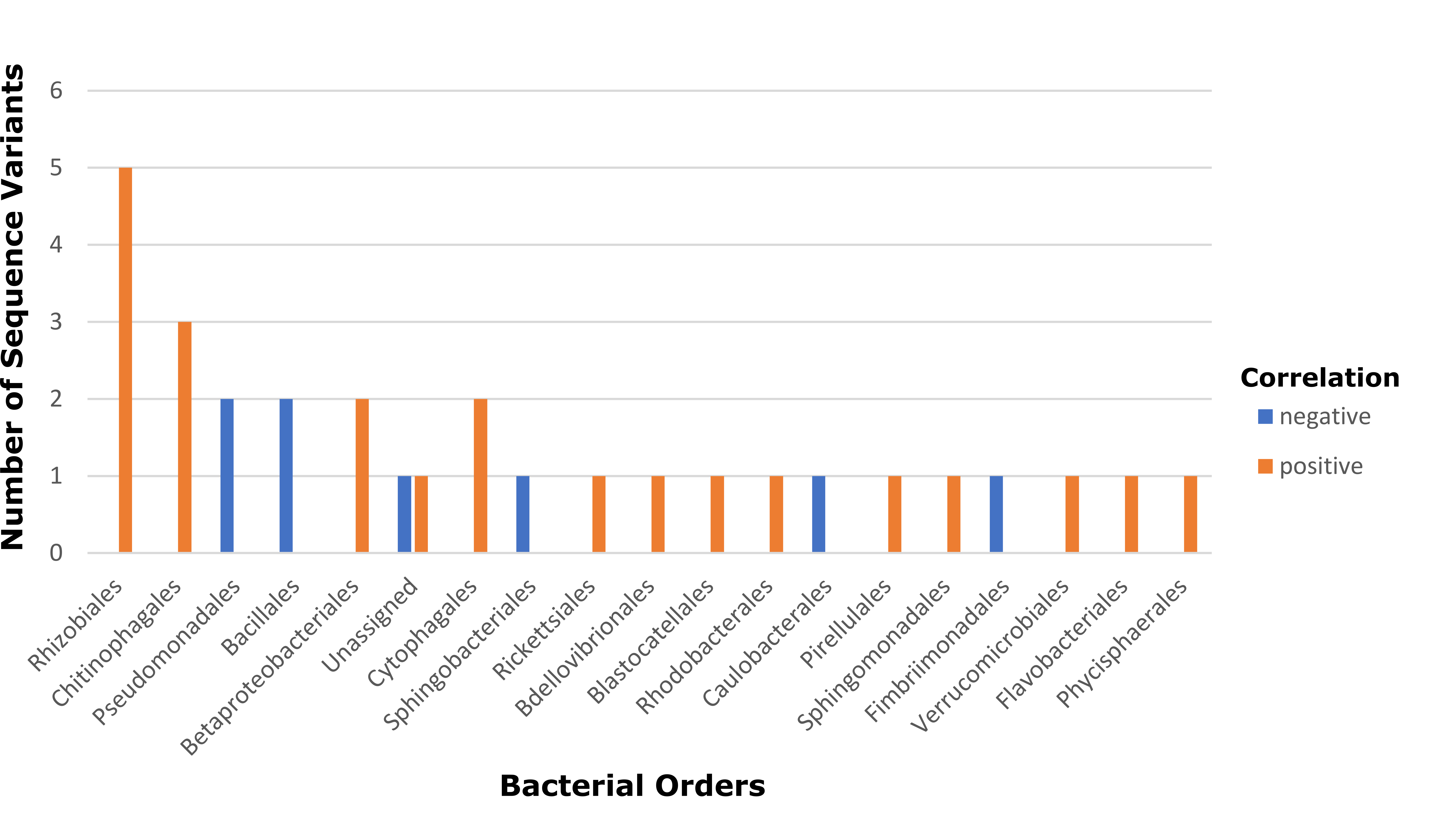

Supplement: Supplementary file 8 — Fig. S8. Bacterial orders most highly correlated with the duration algal cultivation in the outdoor RAFT reactors. Counts of sequence variants with multinomial regression differential values for days after inoculation are indicated by a z score of greater than three. The sequence variants are grouped by their assigned taxonomy at the order‐level, and bars are color‐coded by the direction of the correlation. [file MBT2-13-1546-s008.png]

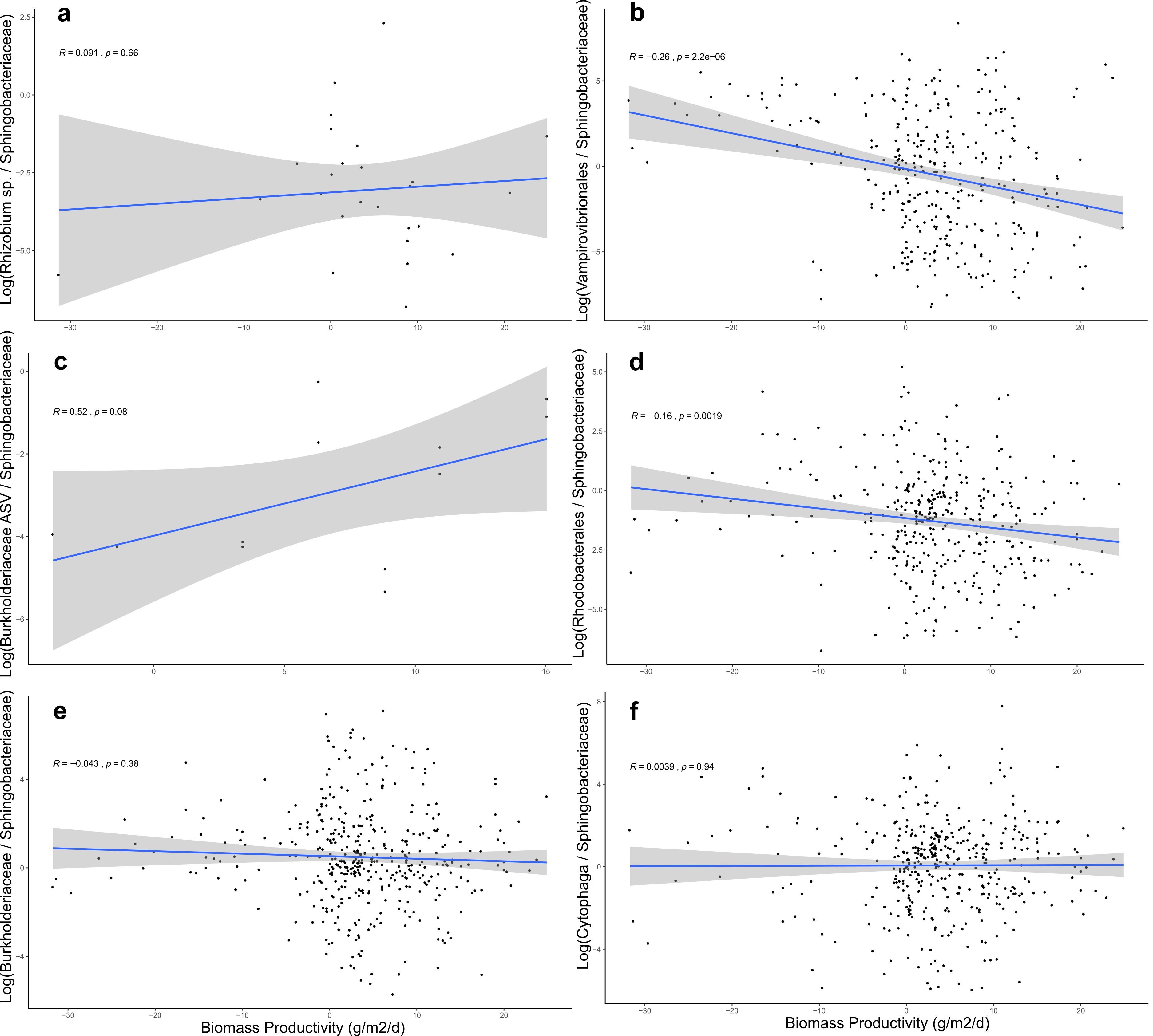

Supplement: Supplementary file 9 — Fig. S9. Log ratio relationships of selected bacterial taxa to algal biomass productivity. The log ratios of the relative abundance of (a) Rhizobium sp. (accession no. JX255399), (b) Vampirovibrionales, (c) Burholderiaceae ASV 21c587e98537024a404d6f041c0fb693, (d) Rhodobacterales, (e) Burkholderiaceae, and (f) Cytophagales to Sphingobacteriaceae for each sample are plotted by their corresponding biomass productivity measure. Lines and grey areas represent linear regression and 95% confidence intervals, respectively, and R fitness and P significance values, based on the Pearson’s correlation test. [file MBT2-13-1546-s009.png]

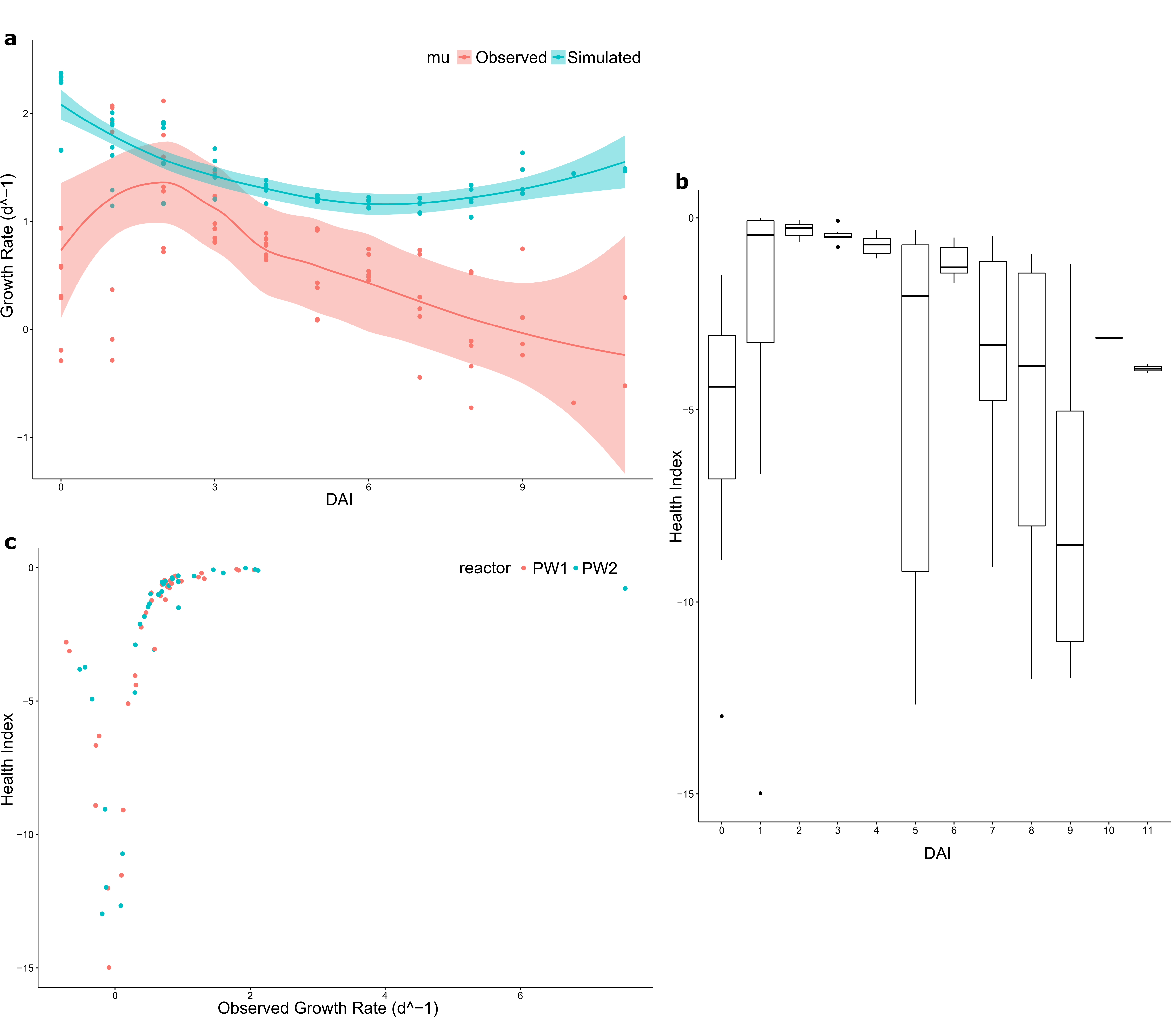

Supplement: Supplementary file 10 — Fig. S10. Comparisons of simulated and observed algal growth rates during RAFT baseline experiments. Observed and simulated algal culture growth rates for all four runs are shown (a) with correlation curves drawn based on loess approximation and 95% confidence intervals indicated by shaded areas. Boxplots (b) indicating the distributions of calculated absolute relative error of growth rates (mu) are displayed by days after inoculation, and the relationship between observed growth rate and calculated absolute relative error of growth rate (c) for the four duplicate runs. [file MBT2-13-1546-s010.png]

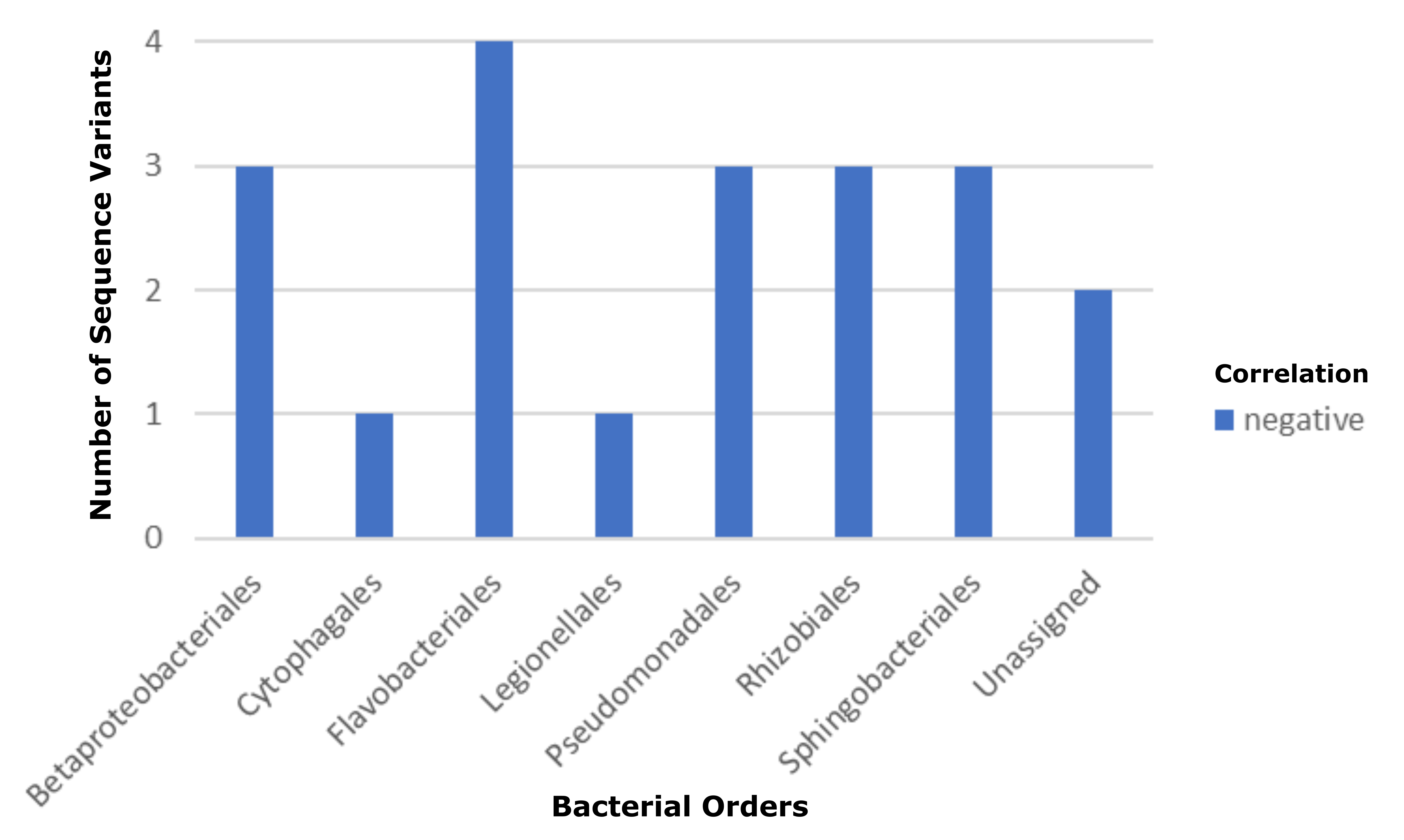

Supplement: Supplementary file 11 — Fig. S11. Bacterial orders most highly correlated with algal Health Index (HI). Counts of sequence variants with multinomial regression differential values for days after inoculation that have a z score greater than three. The sequence variants are grouped by their assigned taxonomies at the order level, and bars are color coded by the direction of the correlation. [file MBT2-13-1546-s011.png]

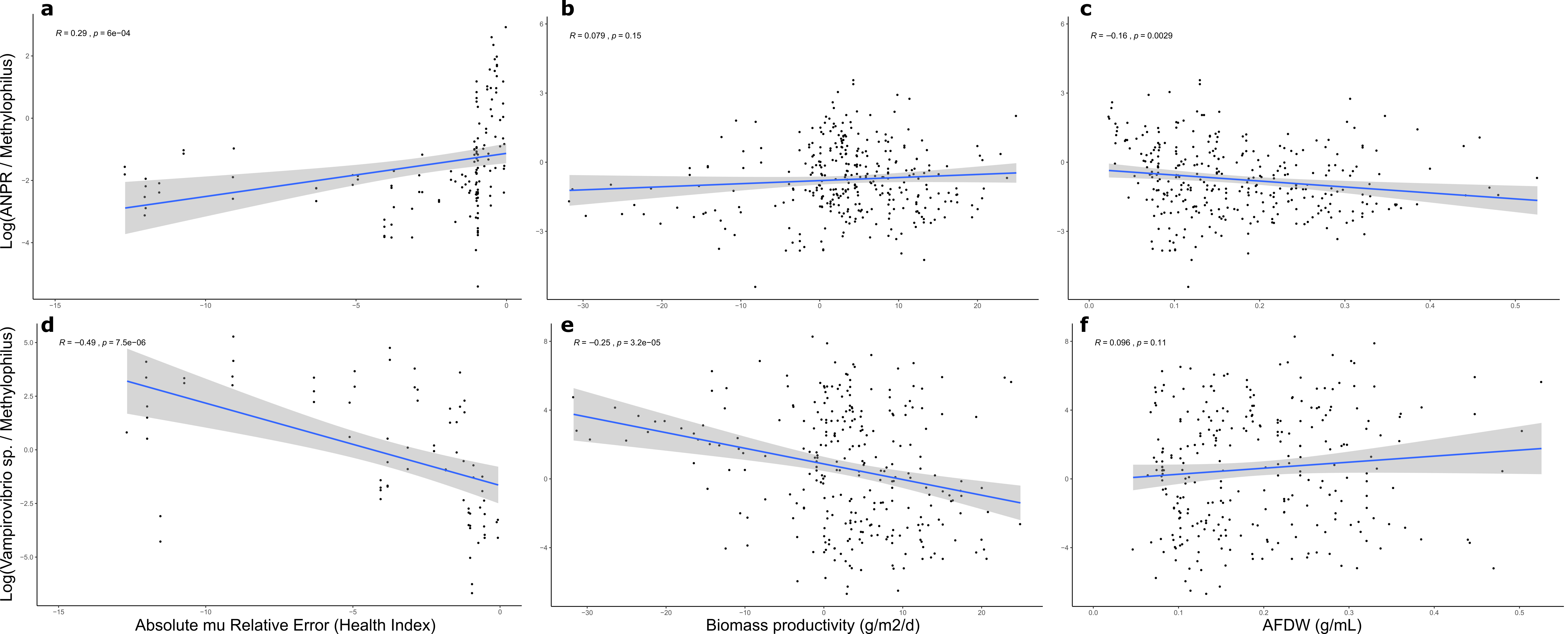

Supplement: Supplementary file 12 — Fig. S12. Comparisons of putative algal culture performance metrics for identification of bacterial interactions. The log ratios of the relative abundance of (a–c) Allorhizobium‐Neorhizobium‐Pararhizobium‐Rhizobium (ANPR) and (d–f) Vampirovibrio sp. to Methylophilus for each sample are plotted by their corresponding algal HI measure, biomass productivity, and ash free dry weight (AFDW). Lines and grey areas represent linear regression and 95% confidence intervals respectively, along with the R fitness and p significance values based on a Pearson correlation test. [file MBT2-13-1546-s012.png]

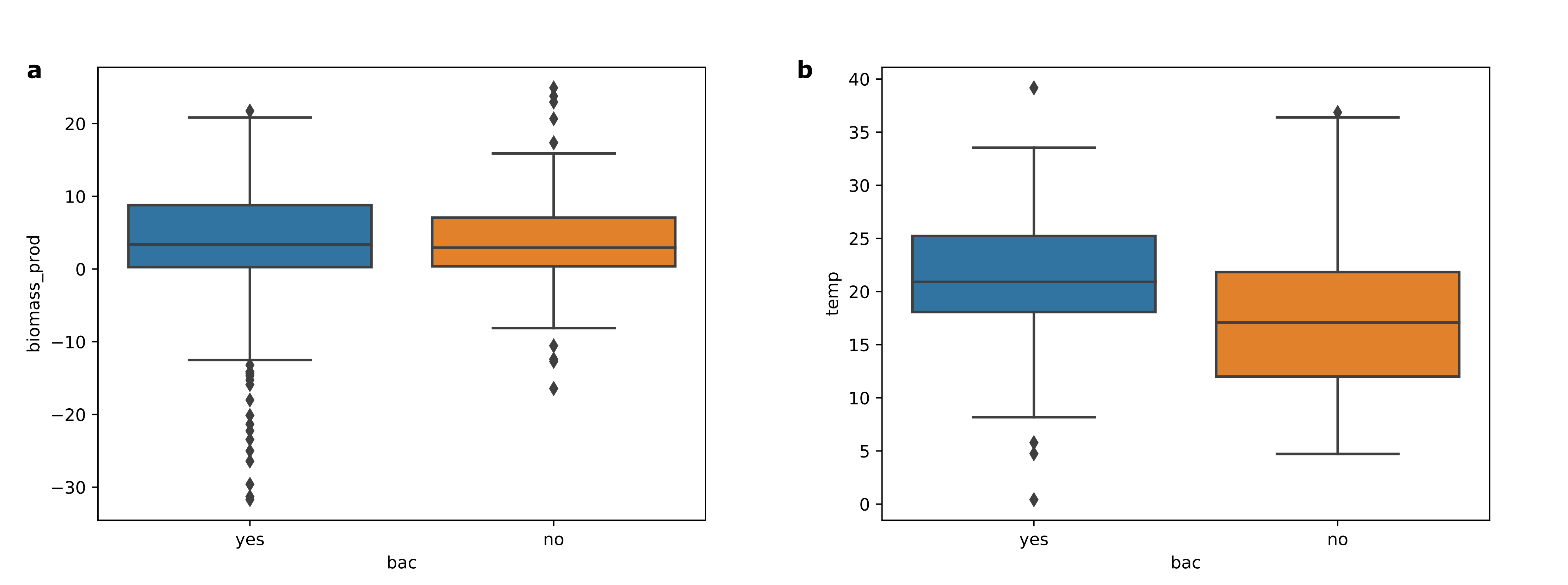

Supplement: Supplementary file 13 — Fig. S13. Distributions of (A) biomass productivity (P = 0.51) and (B) temperature (P = 2.32e−10) measurements for samples collected from cultures treated (blue) or untreated (orange) with benzalkonium chloride. [file MBT2-13-1546-s013.png]
