# Supplementary material for: Association between algal productivity and phycosphere composition in an outdoor Chlorella sorokiniana reactor based on multiple longitudinal analyses
Source: Microb Biotechnol. 2020 May 25;13(5):1546–61. doi: 10.1111/1751-7915.13591 (PMC7415377; doi:10.1111/1751-7915.13591)
Supplement: Supplementary file 14 — Table S1. RAFT baseline experiments continuous data summary. [file MBT2-13-1546-s014.docx]

**Supporting Information Tables**

**Table S1.** RAFT baseline experiments continuous data summary

|  | **Culture temperature (^o^C)** | | |  |  |  |
| --- | --- | --- | --- | --- | --- | --- |
| **Experiment** | **Average** | **Average daily maximum** | **Average daily minimum** | **pH** | **EC (mS/cm)** | **DO (ppm)** |
| R41PW1 | 25.34 ± 1.91 | 32.06 ± 4.01 | 20.67 ± 1.05 | 7.78 ± 0.19 | -0.74 ± 1.2 | 11.78 ± 6.22 |
| R41PW2 | 25.07 ± 1.49 | 30.47 ± 2.77 | 20.9 ± 1.22 | 7.9 ± 0.31 | 0.67 ± 0.22 | 10.76 ± 6.73 |
| R42PW1 | 24.38 ± 1.16 | 30.38 ± 2.08 | 19.78 ± 1.35 | 7.87 ± 0.09 | 0.43 ± 0.24 | 9.12 ± 4.23 |
| R42PW2 | 24.11 ± 1.18 | 30.45 ± 2.67 | 19.83 ± 1.09 | 8.04 ± 0.17 | 0.93 ± 0.18 | 7.36 ± 4.16 |
| R43PW1 | 19.64 ± 2.47 | 25.24 ± 3.57 | 14.37 ± 1.86 | 7.83 ± 0.12 | -1.4 ± 0.6 | 11.52 ± 2.94 |
| R43PW2 | 19.86 ± 2.66 | 25.19 ± 3.56 | 14.51 ± 2.02 | 8.58 ± 0.23 | 0.41 ± 0.39 | 12.18 ± 2.26 |
| R45PW1 | 13.63 ± 1.95 | 18.67 ± 1.98 | 8.33 ± 1.1 | 7.84 ± 0.08 | -2.53 ± 0.33 | 9.6 ± 1 |
| R45PW2 | 13.46 ± 1.98 | 18.6 ± 1.94 | 8.45 ± 2.19 | 7.98 ± 0.06 | 0.74 ± 0.01 | 12.09 ± 1.15 |

**Table S2.** Multinomial model differentials for all amplicon sequence variants and their taxonomic assignments

Attached .xlsx file

**Table S3.** Benzalkonium beta group significance PERMANOVA results

| Distance metric | pseudo-F | p-value | number of permutations |
| --- | --- | --- | --- |
| Jaccard | 14.1213 | 0.001 | 999 |
| Bray-Curtis | 15.2898 | 0.001 | 999 |
| Unweighted unifrac | 15.1213 | 0.001 | 999 |
| Weighted unifrac | 10.3279 | 0.001 | 999 |

**Table S4.** Most significant sequence variants to biomass productivity according to multinomial model

| **Differential** | **Kingdom** | **Phylum** | **Class** | **Order** | **Family** | **Genus** | **Species** |
| --- | --- | --- | --- | --- | --- | --- | --- |
| 5.81 | Bacteria | Proteobacteria | Alphaproteobacteria | Rickettsiales | SM2D12 | unidentified marine bacterioplankton | unidentified marine bacterioplankton |
| 5.47 | Bacteria | Proteobacteria | Deltaproteobacteria | Bdellovibrionales | Bdellovibrionaceae | Bdellovibrio | Bdellovibrio bacteriovorus |
| 2.86 | Bacteria | Proteobacteria | Gammaproteobacteria | Betaproteobacteriales | Burkholderiaceae | Unassigned | Unassigned |
| 2.84 | Bacteria | Proteobacteria | Gammaproteobacteria | Betaproteobacteriales | Burkholderiaceae | Unassigned | Unassigned |
| 2.72 | Bacteria | Bacteroidetes | Bacteroidia | Cytophagales | Cyclobacteriaceae | Algoriphagus | Algoriphagus sp. ZH062 |
| 2.72 | Bacteria | Proteobacteria | Alphaproteobacteria | Rhizobiales | Devosiaceae | Devosia | Unassigned |
| 2.72 | Bacteria | Proteobacteria | Alphaproteobacteria | Rhizobiales | Devosiaceae | Devosia | Unassigned |
| 2.72 | Bacteria | Proteobacteria | Alphaproteobacteria | Rhizobiales | Xanthobacteraceae | Pseudoxanthobacter | uncultured alpha proteobacterium |
| 2.72 | Bacteria | Bacteroidetes | Bacteroidia | Cytophagales | Spirosomaceae | Unassigned | Unassigned |
| 2.72 | Bacteria | Proteobacteria | Gammaproteobacteria | Betaproteobacteriales | Burkholderiaceae | Curvibacter | Unassigned |
| 2.72 | Bacteria | Proteobacteria | Alphaproteobacteria | Rhodobacterales | Rhodobacteraceae | Defluviimonas | Unassigned |
| 2.72 | Bacteria | Proteobacteria | Alphaproteobacteria | Sphingomonadales | Sphingomonadaceae | Unassigned | Unassigned |
| 2.72 | Bacteria | Bacteroidetes | Bacteroidia | Chitinophagales | Chitinophagaceae | Flavihumibacter | uncultured bacterium |
| 2.72 | Bacteria | Planctomycetes | Planctomycetacia | Pirellulales | Pirellulaceae | Rhodopirellula | Unassigned |
| 2.72 | Bacteria | Proteobacteria | Gammaproteobacteria | Betaproteobacteriales | Burkholderiaceae | Hydrogenophaga | Unassigned |
| 2.72 | Unassigned | Unassigned | Unassigned | Unassigned | Unassigned | Unassigned | Unassigned |
| 2.72 | Bacteria | Proteobacteria | Alphaproteobacteria | Rhizobiales | Hyphomicrobiaceae | Hyphomicrobium | Unassigned |
| 2.72 | Bacteria | Acidobacteria | Blastocatellia (Subgroup 4) | Blastocatellales | Blastocatellaceae | uncultured | uncultured bacterium |
| 2.72 | Bacteria | Proteobacteria | Alphaproteobacteria | Rhizobiales | Rhizobiaceae | Unassigned | Unassigned |
| 2.72 | Bacteria | Bacteroidetes | Bacteroidia | Chitinophagales | Chitinophagaceae | Terrimonas | Terrimonas sp. T16R-129 |
| 2.72 | Bacteria | Bacteroidetes | Bacteroidia | Flavobacteriales | Flavobacteriaceae | Flavobacterium | Unassigned |
| 2.72 | Bacteria | Bacteroidetes | Bacteroidia | Chitinophagales | Chitinophagaceae | Parasegetibacter | uncultured bacterium |
| 2.72 | Bacteria | Planctomycetes | Phycisphaerae | Phycisphaerales | Phycisphaeraceae | SM1A02 | metagenome |
| 2.70 | Bacteria | Proteobacteria | Deltaproteobacteria | Oligoflexales | 0319-6G20 | Unassigned | Unassigned |
| 2.70 | Bacteria | Proteobacteria | Deltaproteobacteria | Oligoflexales | Oligoflexaceae | uncultured | uncultured bacterium |
| 2.69 | Bacteria | Proteobacteria | Gammaproteobacteria | Pseudomonadales | Moraxellaceae | Perlucidibaca | uncultured bacterium |
| 2.67 | Bacteria | Proteobacteria | Gammaproteobacteria | Betaproteobacteriales | Burkholderiaceae | Ramlibacter | Unassigned |
| 2.51 | Bacteria | Proteobacteria | Deltaproteobacteria | Bdellovibrionales | Bdellovibrionaceae | Bdellovibrio | Unassigned |
| -2.39 | Bacteria | Proteobacteria | Gammaproteobacteria | Pseudomonadales | Pseudomonadaceae | Pseudomonas | Unassigned |
| -4.65 | Bacteria | Bacteroidetes | Bacteroidia | Cytophagales | Cytophagaceae | Cytophaga | uncultured bacterium |
| -5.40 | Bacteria | Verrucomicrobia | Verrucomicrobiae | Verrucomicrobiales | Rubritaleaceae | Luteolibacter | uncultured bacterium |
| -7.65 | Bacteria | Proteobacteria | Gammaproteobacteria | Oceanospirillales | Pseudohongiellaceae | Pseudohongiella | metagenome |
| -8.03 | Bacteria | Patescibacteria | Parcubacteria | Candidatus Nomurabacteria | Unassigned | Unassigned | Unassigned |
| -8.36 | Bacteria | Proteobacteria | Alphaproteobacteria | Rhodobacterales | Rhodobacteraceae | Unassigned | Unassigned |

**Table S5.** Comparison of bacterial phyla significantly changed by benzalkonium chloride treatment to total amplicon sequence variant taxonomic assignments

| Phylum | Total counts | Total proportion (%) | BAC altered counts | BAC altered proportion (%) |
| --- | --- | --- | --- | --- |
| Proteobacteria | 462 | 50.55 | 66 | 61.68 |
| Bacteroidetes | 209 | 22.87 | 22 | 20.56 |
| Firmicutes | 59 | 6.46 | 1 | 0.93 |
| Verrucomicrobia | 48 | 5.25 | 7 | 6.54 |
| Armatimonadetes | 7 | 0.77 | 2 | 1.87 |
| Unassigned | 37 | 4.05 | 2 | 1.87 |
| Chlamydiae | 10 | 1.09 | 2 | 1.87 |
| Planctomycetes | 21 | 2.30 | 2 | 1.87 |
| Cyanobacteria | 11 | 1.20 | 2 | 1.87 |
| Actinobacteria | 16 | 1.75 | 0 | 0.00 |
| Patescibacteria | 11 | 1.20 | 1 | 0.93 |
| Gemmatimonadetes | 5 | 0.55 | 0 | 0.00 |
| Acidobacteria | 4 | 0.44 | 0 | 0.00 |
| BRC1 | 3 | 0.33 | 0 | 0.00 |
| Deinococcus-Thermus | 3 | 0.33 | 0 | 0.00 |
| Dependentiae | 3 | 0.33 | 0 | 0.00 |
| Spirochaetes | 2 | 0.22 | 0 | 0.00 |
| Tenericutes | 2 | 0.22 | 0 | 0.00 |
| Chloroflexi | 1 | 0.11 | 0 | 0.00 |

**Table S6.** RAFT 16S rRNA gene sample metadata table.

Attached .xlsx file
